# Seizure and redox rescue in a model of glucose transport deficiency

**DOI:** 10.1371/journal.pcbi.1012959

**Published:** 2025-04-04

**Authors:** Jay S. Coggan, Polina Shichkova, Henry Markram, Daniel Keller

**Affiliations:** 1 Blue Brain Project, EPFL: École Polytechnique Fédérale de Lausanne, Geneva, Switzerland; 2 Biognosys AG, Schlieren, Switzerland; Maastricht University: Universiteit Maastricht, NETHERLANDS, KINGDOM OF THE

## Abstract

Disruptions of energy supply to the brain are associated with many neurodegenerative pathologies and are difficult to study due to numerous interlinked metabolic pathways. We explored the effects of diminished energy supply on brain metabolism using a computational model of the neuro-glia-vasculature ensemble, in the form of a neuron, an astrocyte and local blood supply. As a case study, we investigated the glucose transporter type-1 deficiency syndrome (GLUT1-DS), a childhood affliction characterized by impaired glucose utilization and associated with phenotypes including seizures. Compared to neurons, astrocytes exhibited markedly higher metabolite concentration variabilities for all but a few redox species. This effect could signal a role for astrocytes in absorbing the shock of blood nutrient fluctuations. Redox balances were disrupted in GLUT1-DS with lower levels of reducing equivalent carriers NADH and ATP. The best non-glucose nutrient or pharmacotherapies for re-establishing redox normalcy involved lactate, the keto-diet (β-hydroxybutyrate), NAD and Q10 supplementation, suggesting a possible glucose sparing mechanism. GLUT1-DS seizures resulted from after-discharge neuronal firing caused by post-stimulus ATP reductions and impaired Na^+^/K^+^-ATPase, which can be rescued by restoring either normal glucose or by relatively small increases in neuronal ATP.

## Author summary

Neurodegenerative diseases can strike at any age and regardless of the specific symptoms they are inevitably characterized by complex etiologies. One frequent underlying cause involves disrupted brain energy metabolism. Such a disease is GLUT1 deficiency syndrome, which generally afflicts children and results from inhibited expression of the *SLC2A1* gene that codes for a type of glucose transporter. This deficiency hampers the ability of the brain to import and utilize glucose from the blood as an energy supply. Typical clinical presentations of this syndrome include epileptic seizures. The most effective current treatment is the keto diet, which involves severely curtailing carbohydrates and elevating fat consumption, thus enriching the supply of ketone bodies such as β-hydroxybutyrate. In spite of the broad efficacy of this approach, explanations for why it works, as well as the development of alternative pharmacotherapies, continue to elude researchers. Here we tackled the problem with a biologically realistic computational model of the neuro-glia-vasculature unit of energy management in the brain. Our results predict that the seizure phenotype results from after-discharge neuronal firing patterns and can be rescued by restoring normal GLC or ATP balances in neurons. In addition, astrocytes might act and metabolic shock absorbers and redox imbalances associated with GLUT1-DS can be restored with a combination dietary therapy featuring NAD-enhancing and glucose-sparing properties.

## Introduction

Considerable evidence suggests that energy metabolic dysfunctions underlie many neurodegenerative diseases including Alzheimer’s Disease (AD), primary or co-morbid seizures [1–4] (Patel, 2002; Pathak et al., 2013; Sada et al., 2015; Fulton et al., 2021), as well as general age-related cognitive decline [5–7] (Bak et al., 2018; Cunnane et al., 2020; Amorim et al., 2022). Although most observable brain function output is attributed to neuronal activity, the support of neurons by astrocytes and the reliable vascular supply of oxygen and nutrients is essential. This is why the energy management unit of the brain is often referred to as the neuro-glia-vasculature unit (NGV), an oligocellular network that includes a neuron, glia cell and local micro-vasculature [4,8,9] (Magistretti, 2011; Coggan et al., 2018a; Fulton et al., 2021).

While the primary source of energy for the brain is glucose (GLC), the energetic cooperativity in the NGV is anchored by the sharing of resources between the astrocyte and neuron [10]. For example, the astrocyte to neuron lactate shuttle (ANLS) allows astrocytes to supply the neuron with lactate (LAC), a fuel for oxidative metabolism [11,12] (Pellerin and Magistretti, 1994, 2012; Pellerin et al., 1998), although the persistence of this mechanism remains debated [13,14]. ANLS allows neurons to use aerobic glycolysis for additional purposes such as the pentose phosphate pathway (PPP), which is involved in several functions including the glutathione-mediated detoxification of reactive oxygen species (ROS). Other sources of energy for the brain under different conditions are the so-called ketone bodies (e.g., β-hydroxybutyrate (bHB), acetylacetate (AcAc)), as well as lipids via β-oxidation mechanisms and amino acids via conversion to tricarboxylic acid cycle (TCA; Krebs cycle) intermediates that enter the sequence at various anaplerotic entry points.

Feeding the brain begins when circulating blood GLC is taken up across the blood brain barrier (BBB), the endothelial cell layer, across the basal lamina and into the end-feet of astrocytes via GLUT1 facilitative diffusion transporters. GLC then exits the astrocyte into the interstitium and is subsequently taken up into neurons (via the GLUT3 subtype, e.g., Koepsell, 2020) [15]. GLUT1 deficiency syndrome (GLUT1-DS) arises from genetic defects in the expression of the *SLC2A1* gene and is characterized by hypoglycorrhachia (decreased levels of GLC), as well as LAC, in the cerebral spinal fluid (CSF). There is in fact a family of syndromes associated with this genetic mutation with as many phenotypic characteristics, the most common of which is childhood epilepsy [16–20] (De Vivo et al., 1991; Brockmann, 2009; Barros et al., 2017; Koch and Weber, 2019; De Vivo et al., 1973). Although not all epileptic trajectories result from the same etiology, many are associated with metabolic insufficiency such as mitochondrial dysfunction which focuses attention on energy management [4,21,22] (Chen et al., 2010; Fulton et al., 2021; Wesół-Kucharska et al., 2021). Decreased GLUT1 activity also occurs in familiar neurodegenerative diseases such as AD [15,23,24] (Mooradian et al., 1997; Tang et al., 2019; Koepsell, 2020).

Various strategies have been employed to treat GLUT1-DS, including dietary restrictions [24–28] (Jarrett et al., 2008; Klepper, 2008; Gano et al., 2014; Clanton et al., 2017; Tang et al., 2019). A range of abnormal neuronal activities in GLUT1-DS are reversible by diets that often involve ketogenesis [26,29] (Klepper, 2008; Rajasekaran et al., 2022). The ketogenic diet (KD), however, is not universally successful. It is more effective for some seizures than for developmental disorders or dyskinesias [30,31] (Wang et al., 2005; Klepper et al., 2020). The KD also is subject to variable tolerance, low efficacy or failure to achieve adequate ketosis in some patients. Accordingly, alternative therapies have been explored [32] (Hainque et al., 2020). Anaplerosis refers to the entry of TCA cycle metabolites not derived from GLC at different enzymatic steps. Strategies to enhance energy supply with drugs that feed anaplerotic pathways seem theoretically sound, but have provided mixed or disappointing results. [32–35] (Pascual et al., 2014; Mochel et al., 2016; Mochel, 2017; Hainque et al., 2020; Striano et al., 2022).

Less severe phenotypes of GLUT1-DS are also observed, such as paroxysmal exertion-induced dyskinesia, and there are likely to be undiagnosed GLUT1-related conditions [36] (Anand et al., 2011) involving milder GLUT alteration or glycolytic deficiency variants that might underlie other neurological diseases [20] (Barros et al., 2017). This underscores the general utility of studying the effects of impaired GLC metabolism, independently of GLUT1-DS, especially since GLC metabolism is thought to be operating on the edge of criticality [20] (Barros et al., 2017). Even though we focus on GLUT1-DS as a case study, our simulation results should therefore be taken as a general theoretical treatment of the topic.

Many computational models have been constructed to simulate and study NGV behavior in health and disease [9,37–39] (Aubert and Costalat, 2002; Calvetti et al., 2015; Jolivet et al., 2015; Coggan et al., 2018a). Due to the ambiguity in what exactly is happening in the brain metabolically when energy sources are altered, we employ our model to observe the downstream sequelae of reduced GLC importation into the NGV unit by tracking the fates of metabolic species from cytosolic glycolysis through the mitochondrial Krebs cycle or tricarboxylic acid cycle (TCA), as well as the reducing equivalent energy source production of ATP, NADH and NADPH, including from oxidative phosphorylation (OXPHOS). In turn, we examined the effects of various treatments for GLUT1-DS, including a KD, LAC supplementation, or enhanced TCA cycle anaplerosis. Although both are involved, LAC and GLC independently contribute to the developmental and neurological states in GLUT1-DS [40] (Nabatame et al., 2023). There are complex and sometimes paradoxical effects of reduced brain GLC, which supports our assertion that a computational approach is useful for tracking the fates of metabolites [41] (Pascual et al., 2008).

Most dietary therapies, whether carbohydrate-, lipid-rich, or KD, are adapted to address the epileptic phenotype [42] (Pascual and Ronen, 2015). Although KD therapies are the standard of care, they are less effective with age or come with long-term complications and intolerance. The KD can have effects on multiple signaling pathways, including some not directly involved in energy metabolism [27,43] (Gano et al., 2014; Veys et al., 2020), though these aspects are beyond the scope of our model. We also only examine the potential energetic contributions of nutrients to GLUT1-DS and not those related to carbon balance [44,45] (Zilberter and Zilberter, 2020; Avila et al., 2023).

We predict a role for astrocytes in protecting neurons from nutrient fluctuations and identify the best strategies for restoring redox balance when GLC import is impaired. In addition, we propose that seizures associated with GLUT1-DS result from after-discharge firing caused by dysregulated Na^+^/K^+^-ATPase, and that restoration of normal firing can be achieved by elevating local neuronal GLC or ATP.

## Results

### Computational model of GLUT1-DS with effect on selected energy carrying metabolites

**Fig 1:**
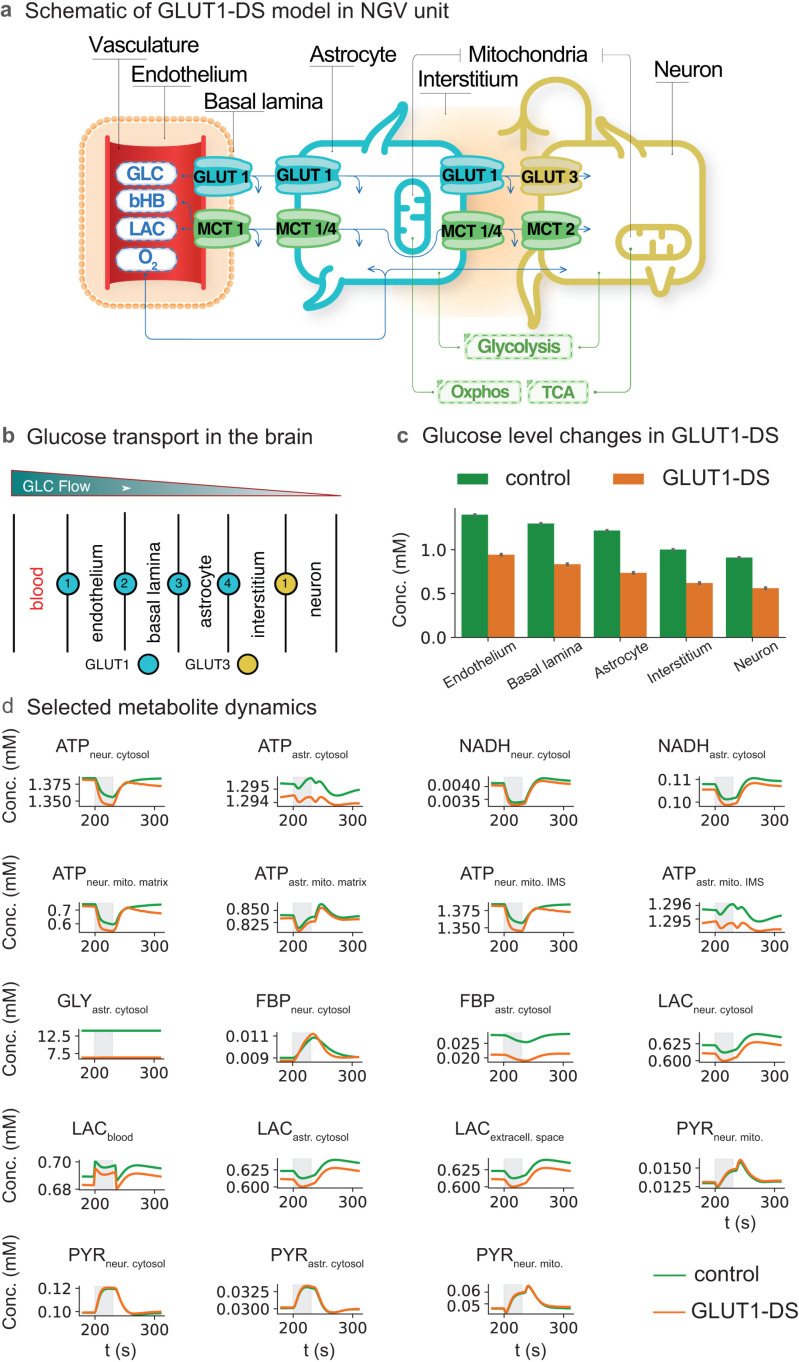
Schematic of model, implementation of GLUT1-deficiency and effects of glucose transport deficiency on selected metabolites. a) sparse schematic diagram of the NGV unit model b) detailed scheme of the use of GLUT1 in 4 membrane partitions between model compartments, and the location of GLUT3 between on the neuron, which remained unaltered in these simulations c) the reduction of [GLC] in various compartments in GLUT1-DS compared to control, GLC in endothelium, extracellular space from blood to astrocyte, GLC in the astrocyte, GLC in the extracellular space between astrocyte and neuron, or interstitium, GLC in the neuron d) time series data for selected metabolites: ATP; NADH; glycogen (GLY, astrocyte only); fructose bisphosphatase (FBP, affected by GLUT1-DS only in the astrocyte), results correspond to the increased sensitivity of FBP in the astrocyte’s preference for glycolytic upregulation; lactate (LAC) in blood (b), astrocyte (a), extracellular space and neuron (neur); cytosolic pyruvate (PYR) and mitochondrial pyruvate (PYRmito) in astrocyte (astr) and neuron (neur). IMS (intermembrane space). Gray shaded area indicates time of neuronal stimulation.

A reduced and stylized schematic diagram of the NGV model gives an overview of the main compartments (one neuron, one astrocyte, one vascular compartment and extracellular spaces) containing the metabolic model components (Fig 1a). A more detailed representation of glucose transport features includes the GLUT1 subtype from the blood (capillary) to endothelium, through basal lamina, astrocyte and interstitium, after which the GLUT subtype changes to GLUT3 in the neuron (Fig 1b). This GLC transport arrangement is based on a previous report [20] (Barros et al., 2017). The concentrations of GLC at different stages of transport between the blood and the neuron, and comparing control (green bars) to GLUT1-DS (orange bars), are shown in Fig 1c.

Time series are presented before, during and after a 30 ms neuronal stimulus of selected metabolites involved in GLC, direct energy delivery (ATP, NADH), storage in the form of glycogen (GLY, astrocyte only), or critical points of regulation (FBP, LAC, PYR), in either the neuron (neur), astrocyte (astr), mitochondria (mito), or extracellular space (Fig 1d).

We compared selected fluxes for redox and energy relevant metabolites (ATP and NADH) in neurons and astrocytes during normal and GLUT1-DS, as well as the ratios of fluxes that are more indicative of redox balance (JATPglyc/JATPox and JNADHglyc/JNADox). In these plots the positive fluxes measured in mM/s represent turnover. Very little change in NADH fluxes was observed for GLUT1-DS in either cell type. ATP fluxes, in contrast, were increased in the neuron cytoplasm, but decreased in the astrocyte cytoplasm. ATP flux increased during and after stimulus in neuronal mitochondria but not astrocytic mitochondria. The sum of ATP fluxes in all compartments increased in the neuron, but remained constant in the astrocyte. Overall, there was a reduction in ATP flux related to glycolysis in the cytoplasm in the astrocyte, resulting in a lower JATPglyc/JATPox during GLUT1-DS, but not the neuron (Fig A in S1 Text).

### Metabolite concentration variability is higher in astrocytes and in GLUT1-DS

In the NGV system, the glia interface directly with the vasculature and are the first brain cell type to absorb nutrients from the blood. GLC, LAC and bHB, for example, must pass through the astrocytes on their way to neurons. This is how the concept of the selfish astrocyte was developed, with the assumption that it would extract its own energy at the expense of the neuron under conditions of scarcity [46] (Barros, 2022). But by the same token, astrocytes are also the first to be exposed to fluctuations in blood metabolite concentrations and form the first line of defense against any dramatic or pathological changes in BBB-permeable blood components.

**Fig 2:**
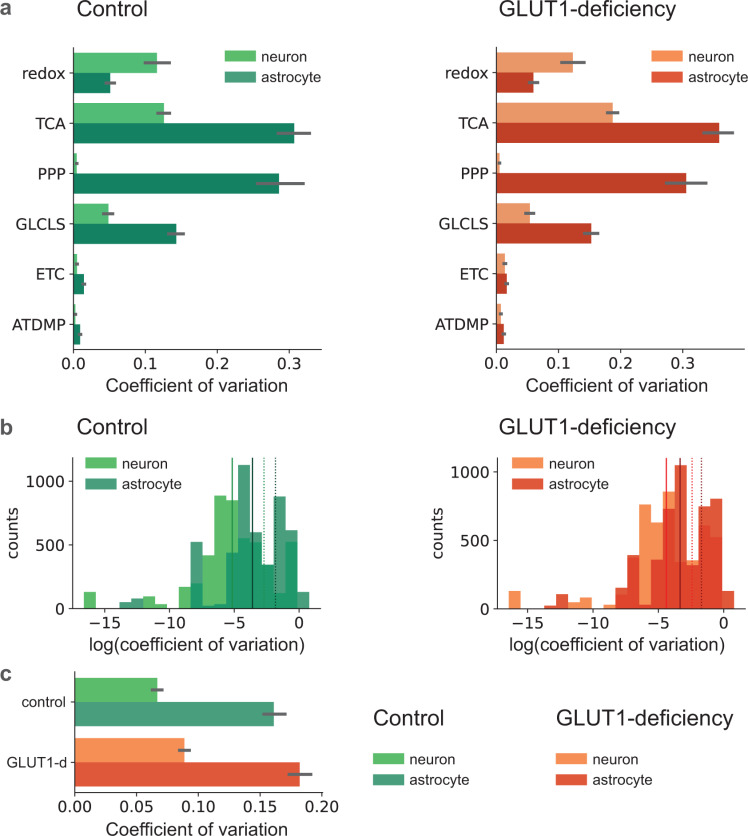
Metabolite concentration variability is higher in astrocytes than in neurons, both in control and GLUT1 deficiency. a) in control simulations (left), and in GLUT1-DS (right), the coefficient of variations (CV) for metabolites for all major subgroups: redox; TCA cycle (TCA), pentose phosphate shunt (PPP), glycolysis (GLCLS), electron transport chain (ETC), and adenosine phosphates (ATDMP). Variations are significantly higher in all groups except the redox molecules (NADH, NADPH, GSH); b) log CV distributions for control (left, green) and GLUT1-DS (right, orange) and comparing neurons (lighter shade) and astrocytes (darker shade), dotted line is the mean of the distribution, solid line is median. Two of each type, neuron and astrocyte; c) side-by-side comparisons show that in addition to higher overall metabolite concentration variability in astrocytes than neurons, there is also a higher variability in the GLUT1-DS condition in both astrocytes and neurons (black bars represent 95% confidence intervals).

We found that metabolite concentration variability is higher in astrocytes than in neurons, both in control and GLUT1-DS. In control simulations, as well as in GLUT1-DS, the coefficient of variation (CV) was higher in the astrocyte for metabolites in most major subgroups: TCA cycle (TCA), pentose phosphate shunt (PPP), glycolysis (GLCLS), electron transport chain (ETC), and adenosine phosphates (ATDMP) (Fig 2a). Notably, defying that trend were redox molecules (NADH, NADPH, GSH) which had lower CVs. The pool of metabolites for control and GLUT1-DS, as well as comparing neurons to astrocytes, showed higher astrocytic log CVs (Fig 2b). Side-by-side comparisons show that in addition to higher overall metabolite concentration variability in astrocytes than neurons, there is also a higher variability in the GLUT1-DS condition in both astrocytes and neurons (Fig 2c).

### Concentrations of metabolic intermediates during GLUT1-DS are largely stable except for certain points of regulation in astrocyte

We looked at the fates of selected energy metabolites associated with specific cellular functions during GLUT1-DS. Not surprisingly for a GLC import impairment disease, we observed significant reduction of GLC in various compartments: GLC in epithelium (Glc_t_t), extracellular space from blood to astrocyte (Glc_ecsBA), GLC in the astrocyte (Glc_a), GLC in the extracellular space between astrocyte and neuron (Glc_escAN), and GLC in the neuron (Glc_n) (Fig B in S1 Text, row 1, panel 1).

We found that concentrations of most metabolic intermediates in GLUT1-DS are largely stable, except for certain points of regulation in astrocytes. Notably, at fructose bisphosphatase (FBP), the increased sensitivity of FBP in the astrocyte corresponds to its responsiveness to glycolytic upregulation. Also more unstable than the majority of metabolites were dehydroxyacetone phosphate (GAP) and glyceraldehyde-3-phosphate (DHAP), which are also central points of regulation for the astrocytic preference for glycolysis, in addition to being involved in methylglyoxal production [47] (Allaman et al., 2015) (Fig B in S1 Text). It is easy to speculate that the increase in sensitivity of these three metabolites is related to their situation at pathway regulatory nodes, where their role would be to adjust metabolic pathways according to resource availability.

LAC was not significantly affected by GLUT1-DS in all compartments: blood LAC (Lac_b), extracellular LAC (Lac_ecs), astrocyte LAC (Lac_a), and neuron LAC (Lac_n). Neither were there any notable effects of GLUT1-DS on points of anaplerotic regulation: oxaloacetate (OXAmito), succinylCoA (SUCCOAmito), a-ketoglutarate (AKGmito), or fumarate (FUMmito). Other TCA metabolites not affected in either neuron or astrocyte included acetylCoA (AcCoAmito), coenzymeA (CoAmito), isocitrate (ISOCITmito), pyruvate (PYRmito), citrate (CITmito), malate (MALmito), succinate (SUCmito) (Fig B in S1 Text).

### Recovery of redox balance by dietary or blood supplementation therapy

The inhibition of GLC import into cells could have an impact on the relative contributions of glycolytic (cytosolic) energy production vs TCA and the ETC. In this context, we examined the bioenergetic scope of the neurons and glia in response to GLUT1-DS. Bioenergeic scope refers to a cell’s flexibility in response to changes in demand or supply of energy [48] (Mookerjee et al., 2017). In our case the energy production location would potentially change in response to reduced GLC import. For example, this change might be reflected in shifts between cytosolic and mitochondrial compartments.

**Fig 3:**
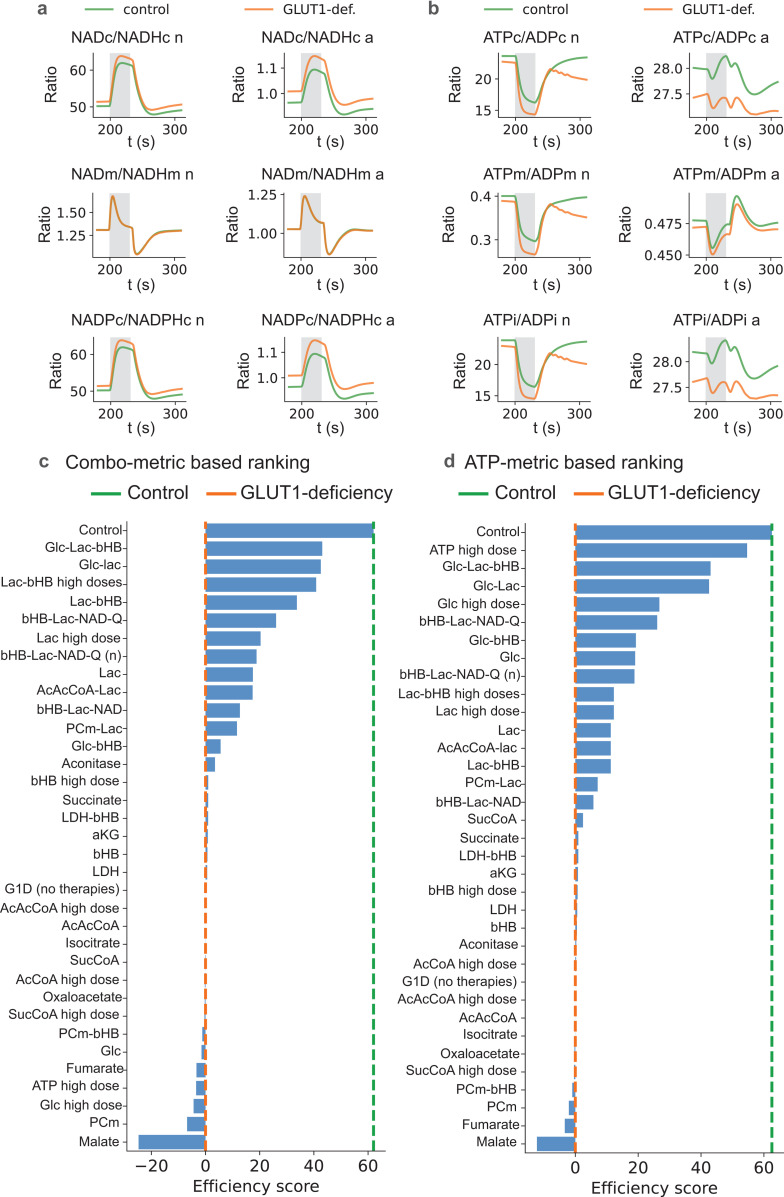
Redox ratios and ranking of potential therapies by efficacy to restore redox balance. a) The ratios in neurons (left column) and astrocytes (right column), for NAD^+^/NADH in cytoplasm (upper row) or mitochondria (middle row), and NADP^+^/NADPH (bottom row). GLUT1-DS had no effect on mitochondrial NAD^+^/NADH in either cell type, but greater effect in the astrocytes for both cytoplasmic NAD^+^/NADH and NADP^+^/NADPH, b) The ATP/ADP ratios in neurons (left column) and astrocytes (right column), in cytoplasm (upper row), mitochondrial matrix (middle row) and intermembrane space (bottom row). GLUT1-DS reduced these ratios in all cases, but again had the largest effect on cytplasmic levels in the astrocyte. The effects in the neuron were notably pronounced in the post-stimulus phase. Overall, the effects of GLUT1-DS on redox rations in the neuron and astrocyte are opposite. c) the ranking of the efficacy of therapies to restore total redox ratio balance (NAD^+^/NADH + ATP/ADP + NADP^+^/NADPH. Best therapy is Glc-lac-bHB. d) the ranking of the efficacy of therapies to restore total ATP/ADP balance only. Best therapy is ATP high-dose, but the best reasonable therapy without ATP (which cannot be easily done with just dietary supplements, and without extra GLC added to diet, which can cause health problems for some people, is the bHB-LAC-NAD-Q therapy).

We plotted the redox ratios of the NAD-related energy carrying molecules in neurons and astrocytes, in their respective cytoplasmic and mitochondrial compartments, and in both normal and GLUT1-DS conditions, including cytosolic NAD^+^/NADH (Fig 3a, top row), mitochondrial NAD^+^/NADH (Fig 3a, middle row), NADP^+^/NADPH in cytoplasm (Fig 3a, bottom row). The results indicated an increase in the cytoplasmic NAD^+^/NADH and NADP/NADPH ratios during GLUT1-DS in both neurons and astrocytes, with a stronger effect in the latter, while no effect of GLUT1-DS was seen in mitochondria for either cell type. Similarly, we examined the ratios of ATP/ADP in cytoplasm, mitochondria matrix and intermembrane space (Fig 3b, upper, middle and lower panels, respectively). In all categories, ATP/ADP declined in all measured compartments during GLUT1-DS, but again had the largest effect on cytoplasmic levels in the astrocyte. Of special note is the decline in the ATP/ADP index in the neuron in the post-stimulus period. This effect will prove to be of great significance in the next section. Altogether, the effects of GLUT1-DS on redox ratios in the neuron and astrocyte went in opposite directions.

Since diet is the primary focus of GLUT1-DS treatment, as well as being of general interest for other diseases with suspected association with redox balance in the brain, the next step in our analysis was an attempt to restore normal levels of redox equivalents in the overall NGV (NAD^+^/NADH, ATP/ADP, and NADP^+^/NADPH) with various supplemental nutrient therapies (see S1 Text for procedure details). We ranked the potential of these therapies by their efficacy in restoring the sum of redox equivalents balances (NAD^+^/NADH + ATP/ADP + NADP^+^/NADPH). The best therapy for the total redox restoration was GLC-LAC-bHB. But when solving only for ATP restoration, the ranking of the efficacy of therapies was highest for ATP high-dose, but the best reasonable therapy without ATP (which cannot be easily done with just dietary supplements, and without extra GLC added to diet, which can cause health problems for some people), was the bHB-LAC-NAD-Q therapy. In our model, NAD represents injection of NAD^+^ into cells computationally, which in reality could reflect either extra tryptophan consumption or other dietary supplementations. The amino acid tryptophan is often used as a dietary supplement that can serve as a precursor for various neuroactive substances, typically serotonin, but also NAD^+^ through the kynurenine system [49] (Castro-Portuguez and Sutphin, 2020).

Coenzyme Q10 therapy, referred to simply as Q in this paper, is also added directly to cells. Q is ubiquitous in the body including in neurons and glia. Depleted levels of Q can restore cultured human neuronal cells [50] (Duberley et al., 2014). Furthermore, the age-related risk of developing neurological disorders tracks with the age-related depletion of tissue Q levels [51] (Mantle et al., 2021).

### Energy metabolite concentrations during GLUT1-DS and optimal therapy

We looked at all of the responses of energy cascade metabolites in the cytoplasm and mitochondria for neurons and astrocytes in control, GLUT1-def and the GLC-LAC-bHB highest rank therapy for redox recovery (Fig C in S1 Text). Most of the results showed broad metabolite stability during GLUT1-DS. Some changes in response to the disease state reassured our understanding of the disease, such as the reduction in GLC in all compartments, and the heightened recovery of GLC and LAC during the GLC-LAC-bHB therapy. The reductions in astrocytic GAP, DHAP and FBP mentioned above (Fig B in S1 Text) were also accompanied by unusually high recovery levels during the GLC-LAC-bHB therapy.

### Relative changes in energy metabolite levels in GLUT1-DS and during therapy

We compared relative changes in metabolite levels in control and GLUT1-DS in the four model compartments (neuron, astrocyte, blood, extracellular space), and clustered the metabolites by metabolic functional groups: ketogenic diet (keto), redox metabolites (redox), respiration excluding electron transport chain (resp), electron transport chain (ETC), adenosine phosphates (ATDMP), glycolysis (GLCLS), TCA cycle (TCA), glutamate-glutamine cycle (GLTGLN), pentose phosphate shunt (PPP). A heatmap summarizing these changes provides a better visualization of which metabolites are going up or down under different conditions. We implemented such a map with average clustering with cosine metric, comparing relative changes in metabolite levels in control and GLUT1-DS in the compartments (Fig D in S1 Text).

Overall, we observe that glucose transport and glycolysis metabolites are at higher levels in control, while some of the therapies boost mitochondrial metabolism and ATP production in GLUT1-DS conditions. It is worth noting that the therapies don’t fully “restore” the GLUT1 deficient system to the control state if we consider all metabolites together. Instead, other states with a similar molecular phenotype are found.

### Lactate fluxes describe the role of ANLS during GLUT1-DS and during treatment

**Fig 4:**
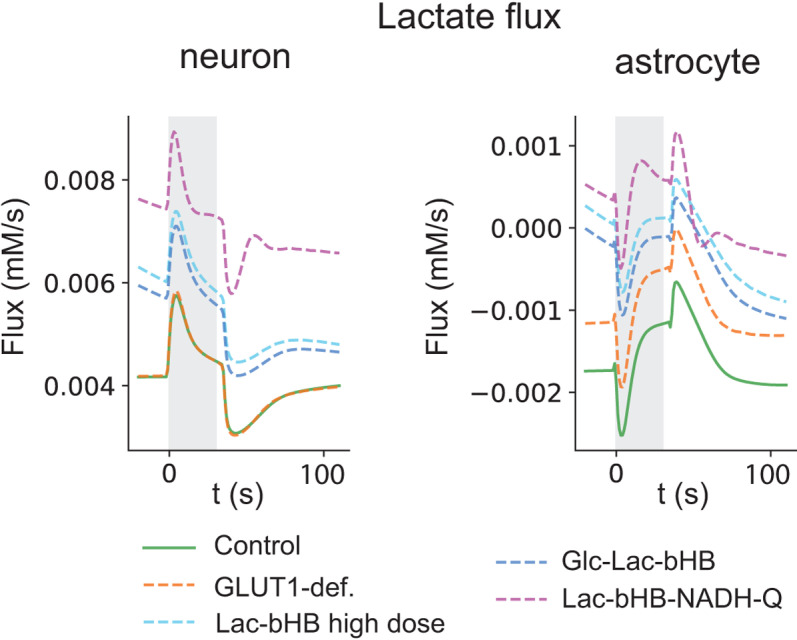
Lactate fluxes describe the role of ANLS in control condition, in GLUT1-DS and during treatment protocol. In the neuron (left panel) and astrocyte (right panel), LAC fluxes were elevated in all treatment conditions compared to control, suggesting a role for increased LAC availability in treatment success outcomes. Non-negative fluxes in both neuron and astrocyte during treatments results from increased LAC production from dietary supplementation.

Since ANLS likely plays a central role in the energy regulation of the NGV, we tested the effects of: 1) the two LAC-based treatments, 2) the two best restorative redox therapies for GLUT1-DS, 3) the best overall therapy that includes GLC (GLC-LAC-bHB), and 4) the best therapy that does not include GLC (LAC-bHB-NAD-Q). We looked at LAC fluxes to describe the role of ANLS in the control condition, during GLUT1-DS and during treatment protocols. In the neuron (Fig 4, left panel) and astrocyte (Fig 4, right panel). LAC fluxes were elevated in all treatment conditions in both astrocytes and neurons compared to control, suggesting a role for increased LAC availability in treatment success outcomes. Non-negative fluxes in both neuron and astrocyte during treatments results from increased LAC production from dietary supplementation. Since these are fluxes elicited by dietary supplementation, the astrocyte will also take up LAC from the blood supply. There was no difference between control and GLUT1-DS in the neuron, but with treatment conditions there was an increase in LAC flux, culminating in the LAC-bHB-NADH-Q combination therapy. In parallel, the astrocyte experienced increased flux in the disease condition. This result could mirror the higher metabolite variability associated with astrocytes (above, Fig 2).

### Afterdischarge-related seizure induction by GLUT1-DS and recovery by exogenous GLC and ATP

Seizures frequently accompany many energy metabolic dysfunctions including GLUT1-DS [16,21,52] (De Vivo et al., 1991; Patel, 2004; Chen et al., 2010), but the mechanisms can be varied or poorly characterized [53] (Kovács et al., 2018). The term “after-discharge” refers to neuronal electrical excitation that continues beyond the duration of a stimulus. When occurring in the cortex, these rogue action potentials (APs) are often associated with seizures [54] (Gollwitzer et al., 2018).

**Fig 5:**
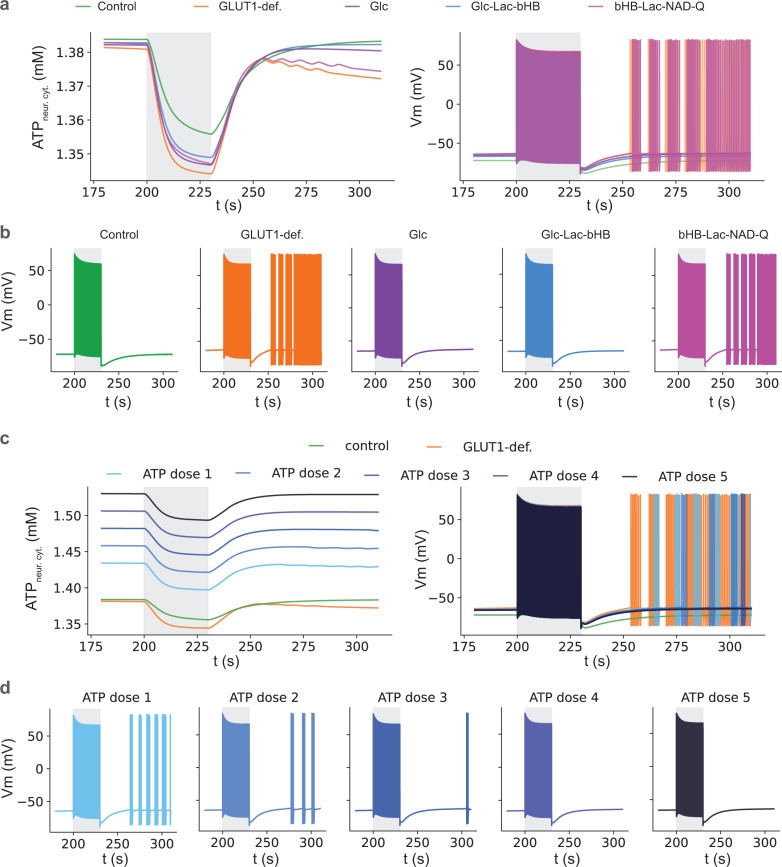
The seizure phenotype in the GLUT1-DS model results from after-discharge firing, and can be rescued by either GLC or ATP. a) time series of ATP in the neuron (left panel) and voltage traces (right panel) during control (green), GLUT1-DS (orange), glc (purple), glc-lac-bHb (blue), and bHB-lac-NAD-Q therapy (purple). Note the saw-tooth pattern in the afterdischarge trace, which corresponds to neuronal firing patterns, is eliminated by glc and glc-lac-bHb therapy. b) separated individual voltage traces from panel a, right, for clarity. c) time series of ATP in the neuron (left panel) and voltage traces (right panel) for control, GLUT1-DS and 5 doses of ATP. d) separated individual voltage traces from panel c, right, for clarity. Note that as the dose of ATP increases, the number of afterdischarge APs reduces until the seizures are completely gone at dose 4. Gray shaded area indicates time of neuronal stimulation.

The seizure phenotype in our GLUT1-DS model resulted from after-discharge firing. GLUT1-DS caused a reduction in ATP, especially in the stimulus recovery time period (Fig 5a, left panel), which in turn triggered after-discharge neuronal spiking that could be rescued by GLC-containing treatments (GLC, GLC-LAC-bHB), but not by bHB-LAC-NAD-Q even though it ranked high in redox rescue simulations from the previous section (Fig 5a, right panel). Separated time series of firing patterns for these groups are shown (Fig 5b). Note that the saw-tooth pattern in the after-discharge traces, which corresponds to neuronal action potentials, is also eliminated by GLC and GLC-LAC-bHb therapy.

In addition to GLC, the after-discharge seizures in our model were also abated by supplemental ATP in the neuron. Time series of control, GLUT1-DS, and application of 5 incremental doses of exogenous ATP effectively raised neuronal concentrations of ATP (Fig 5c, left panel). This theoretical treatment was also able to overcome the GLUT1-DS-associated after-discharge firing (Fig 5c, right panel). Separated individual voltage traces are displayed for clarity (Fig 5d). Note that as the dose of ATP increases, the number of after-discharge APs reduces until the seizures are completely gone at dose 4.

## Discussion

We analyzed the effects of GLUT1-DS on brain energy metabolism with a biologically realistic computational model of the NGV [55] (Shichkova et al., 2025). The model revealed three primary new insights into brain energy regulation. The first observation is that a GLUT1-DS associated ictal phenotype results from after-discharge neuronal firing patterns caused by Na^+^/K^+^-ATPase rundown, and these seizures can be rescued by restoring normal GLC or ATP balances in neurons. The second insight is that redox imbalances associated with GLUT1-DS that are characterized by reduced baseline NADH, NADPH and ATP, in addition to a reduction in energy produced by glycolysis in the neuron. These redox balances can be restored with selected dietary therapies involving GLC, LAC, and KD, or for non-hyperglycemic diets, a LAC, KD, NAD and ubiquinone supplemental combination therapy. The third insight is that astrocytes are likely to buffer metabolite concentration swings resulting from nutritional inconsistencies with markedly higher variabilities than in neurons.

### Epilepsy

Epilepsy is a frequent phenotype in numerous neurodegenerative diseases, although the mechanisms can be unclear or non-unique [56,57] (Coyle and Puttfarcken, 1993; Melov et al., 1999). We suggest a mechanism for neuronal hyperexcitability in GLUT1-DS that can exist independently of several factors thought to be involved in other ictal types, such as glycogen depletion [58] (Brown et al., 2003) or inhibitory interneuron dysfunction [29] (Rajasekaran et al., 2022). The after-discharge firing responsible for the seizures observed in our model has been associated with bistable ionic balance shifts [54,59,60] (Coggan et al., 2010; Baier et al., 2017; Gollwitzer et al., 2018).

The role of ATP in seizure regulation can be complicated by various factors that play a role *in vivo* [61,62] (Ye et al., 2015; Jirsa et al., 2017), such as purine receptors or ATP-dependent K^+^ channels. In rat experiments, for example, antagonism of ATP-sensitive purinergic receptors was shown to reduce epileptogenesis as well as seizure severity [63] (Beamer et al., 2019). Even though the epileptic phenotype is very complex, multi-factorial and affected by comorbidities, our results indicate that at least one simple cause of afterdischarge-related epilepsy can be described by the connection of a dysregulated Na^+^/K^+^-ATPase to simple Hodgkin-Huxley action potential models, since our model exclusively incorporates the role of ATP and GLC in the regulation of Na^+^/K^+^-ATPase activity.

### Therapeutic strategies

Our model’s rescue strategies roughly corresponded to 3 of the 6 brain energy rescue strategies outlined by Cunnane and colleagues [6], including ATP and redox support, GLC or aerobic glycolysis enhancement via LAC, ketosis, and TCA enhancement via anaplerosis. The ATP support, GLC and LAC supplementation turned out to have the strongest effects. The model results offer a new take on the therapeutic mechanism of the KD, which is thought to be effective for GLUT1-DS because it supplies ketone bodies as energy to the brain and has antiepileptic properties [31] (Klepper et al., 2020). Although used extensively in the diet and metabolism literature, the definition of ketosis is weakly quantitative (partially due to patient compliance issues) and it remains to be shown how seizure relief corresponds to the extent of blood keto levels [61,64,65] (Ye et al., 2015; Zilberter and Zilberter, 2018, Ramm-Pettersen et al., 2014).

The effectiveness of the KD to treat the seizure symptoms in our model was weak. One possible explanation is that the time to go into ketosis after fasting is only 2–4 days. But it has been observed that the amelioration of epilepsy by the keto diet takes from days up to weeks and is most successful with early treatment initiation [66] (Schwantje et al., 2020). This discrepancy suggests that the delay to achieve seizure rescue by KD might also require cellular changes that take longer to effect, such as up or down regulation of proteins, and not just alternative energy sources. The faster response of seizures to the KD in early diagnosed patients could also argue the same point; longer time constant cellular changes may begin to occur upon GLUT1-DS onset and the sooner the course of these changes can be interrupted, the faster the KD will work.

Simple alternative treatments involving timed GLC and exercise for paroxysmal non-epileptic events have been proposed that suggest GLC and LAC together could be part of a tailored, non-keto solution for some manifestations of GLUT1-DS [67] (Mortaji et al., 2023). Our results support this hypothesis (with the acknowledgement that GLC supplementation carries its own risks). In the same vein, the combination of both LAC and KD has also been probed to treat TBI and other neurodegenerative conditions [68] (Omori et al., 2022). Our results suggest that GLUT1-DS patients might benefit from such treatments. However, the KD might be preferable to LAC supplementation due to adverse global effects of excessive blood LAC. One of the effects of KD more localized to the NGV is increased local astrocytic export of LAC [69] (Zhang et al., 2022). In this way, the KD might increase the production and availability of LAC for energy use in the NGV, not only to provide bHB and AcCoA to the TCA (the standard mechanism).

Experimental data show that brain bHB can rise significantly in plasma during fasting-induced ketosis in children. LAC elevation by the KD may contribute to KD efficacy in GLUT1-DS. A concomitant LAC increase likely results from ketones displacing LAC oxidation without altering GLC phosphorylation and glycolysis [70] (Pan et al., 2000). Our results indicate that bHB alone is not sufficient to attenuate the seizure phenotype expressed in our model, suggesting the KD has effects on pathways not included in the model. Alternatively, the better efficacy of LAC combined with bHB in supporting normal redox states supports the idea that one of the mechanisms of bHB involves increased local LAC availability. Our model also failed to show any ameliorative effects of injecting metabolites at points of anaplerotic entry into the TCA cycle, a result which supports recent clinical findings that triheptanoin is ineffective in reducing seizures in patients with GLUT1-DS who are not already on a KD diet [35].

Cerebrospinal glucose (CSFG) and lactate (CSFL) both play important roles in motor development and control and CSFL may also contribute to neurological status independently of CSFG [40] (Nabatame et al., 2023). This implies that LAC and GLC contribute to predicting neurological function through independent mechanisms, although whether ANLS [8] (Magistretti, 2011) is directly involved in GLUT1-DS symptoms is unclear. In any case, increased CSFL in patients with GLUT1-DS appears to be beneficial [40] (Nabatame et al., 2023). It is not surprising that both GLC and LAC are part of an optimal redox restoration package. Much experimental evidence points to both sources of energy for active neurons with LAC possibly contributing half or more [14,71,72]. Although controversies remain about the ANLS [13,73], there is considerable evidence supporting this energy supply mechanism [74,75].

There is already a precedent for treatments involving both LAC and KD for neurodegenerative diseases and brain injury, conditions which also can be associated with seizures and decreased ATP [68,76] (Lilamand et al., 2022; Omori et al., 2022). Our results are consistent with this approach but indicate that NAD^+^ and ubiquinone (Q) therapies result in even more optimal restoration of energy substrates. Recently, LAC infusions have been successfully employed to treat patients with hypoglycemia, TBI and Alzheimer’s, suggesting that GLUT1-DS patients resistant to the KD might also benefit from this treatment [77] (van Gemert et al., 2022).

Combination therapies have been tested that attempt to solve both the energy and carbon deficits associated with GLUT1-DS [45] (Avila et al., 2023). It must be emphasized that our approach to combination therapy is restricted to the energy deficit phenotypes which are also sufficiently diverse that they may require a cocktail therapy for optimal results.

### Redox balance

Central to the dynamics of cellular energy metabolism are the production of the energy carrying molecules by way of energy rich phosphate or hydrogen bonds (ATP, GTP, NADH, NADPH, FADH_2_). Generally, ATP and NADH are the main focus since they carry the most energy charge. Thus, the ratios of ATP/ADP and NAD^+^/NADH are a proxy for the energy state of the cell and can be affected by therapies or diets [78] (Elamin et al., 2017). In addition, many researchers place an emphasis on how much of the energy is extracted from GLC by glycolysis in the cytoplasm vs. the TCA cycle and oxidative phosphorylation (aka, the electron transport train) in the mitochondrion. This breakdown is often referred to as the glycolytic index [79] (Barros et al., 2021). The sum of ATP fluxes in all compartments increased in the neuron, but remained constant in the astrocyte. Overall, there was a reduction in ATP flux related to glycolysis in the neuronal cytoplasm, resulting in a lower JATPglyc/JATPox in the neuron in GLUT1-DS, but not the astrocyte. This suggests the neuron suffers energetically more from GLC depletion than does the astrocyte, and is forced to rely more on OXPHOS in GLUT1-DS for ATP supply.

Our optimal therapy results for redox recovery support the role of GLC sparing, not only for non-energetic roles of GLC [44] (Zilberter and Zilberter, 2020) but for efficient glycolysis as well. Even if neurons benefit from metabolic support from astrocytes, for example through ANLS, the significant dependence of neurons on GLC is not precluded [80] (Li et al., 2023). But the inclusion of GLC along with bHB and LAC goes counter to other dietary effects, such as the obesogenic consequences of both a high fat and high carbohydrate diet [81] (Zilberter, 2011), as well as the risk of neuropathy [82] (Yagihashi et al., 2011). Hyperglycemia also promotes oxidative stress and neurodegeneration [1,76,83] (Gibson, 2002; Patel, 2002; Lilamand et al., 2022). As such, increasing the availability of GLC in the neuron either by LAC and bHB or slowing down glycolysis by some other means (GLC sparing) is preferable. The availability of LAC to the neuron scales linearly with the blood concentration, making LAC a reasonably safe and controllable option [72].

### Metabolite variability

The greater variability of metabolites in the astrocyte as compared to the corresponding neuron suggests that glia might absorb some of the metabolic shocks resulting from highly variable blood nutrient content, a function that might subserve its already well-known role in regulating brain energy metabolism [84] (Beard et al., 2021). This observation and our interpretation of the meaning assumes that any metabolic pathways or anatomical features missing from our model would not dampen this effect. We do not think that is likely considering the very similar pathways and anatomical complexity of the corresponding neuron.

One specific and surprising observation was the significant reduction in GAP and DHAP in the astrocyte, but notably not the neuron, in GLUT1-DS. Experimental reports show that GAP and DHAP are the sources of methylglyoxal production in cells that favor glycolysis [85] (Richard, 1993), which is the case for astrocytes [86] (Almeida et al., 2002). These molecules also can serve as a link to lipid metabolism. While our model does not include the glyoxylase system *per se*, the increased responsivity of astrocytes at the level of DHAP and GAP when the GLC supply is reduced could reflect how astrocytes are “wired” more specifically to modulate glycolysis. A potential reduction in methylglyoxyl production could also result in a reduction in astrocytic LAC formation which could affect ANLS [47] (Allaman et al., 2015).

### Perspective and conclusions

The primary finding of our NGV metabolic model of GLUT1-DS is the prediction that one possible mechanism of the clinically observed seizure phenotypes is after-discharge firing, which can be rescued by restoring normal Na^+^/K^+^ ATPase function with either GLC or relatively small increases in neuronal ATP. Achieving the same effect with the KD alone was less effective. But, given that KD is so effective in children, the implication is that our model is not capturing an important feature of the seizure phenotype, the downstream effects of KD, or additional seizure mechanisms present in GLUT1-DS. Given that we show how KD might exert its influence by LAC or GLC sparing, it could also simply be that the model is not sensitive enough to these downstream effects of KD.

ATP levels are remarkably stable in neurons, seemingly for a variety of reasons including proteostasis [87] (Takaine et al., 2022), its function as a hydrotrope for the stabilization of soluble proteins [88] (Patel et al., 2017) and the buffering of Pi. Our results showed that ATP can remain relatively stable in the NGV yet be closely tuned to neuronal Na^+^/K^+^ ATPase. This observation is consistent with reports that mitochondrial cytosolic ATP levels remain stable across a wide range of workloads, with ATP serving as a flux coupler between the Na^+^/K^+^-ATPase and mitochondria [89] (Baeza-Lehnert et al., 2019). The reliance of neurons on adequate Na^+^/K^+^-ATPase function is not surprising considering the large fraction of energy that is dedicated to restoring Na^+^ and K^+^ gradients after neuronal activity [90] (Harris et al., 2012). With these observations, we can predict with some justification that small baseline reductions in neuronal ATP, and subsequent Na^+^/K^+^-ATPase rundown, are sufficient to produce a seizure phenotype due to after-discharge firing.

A computational model can be an indispensable tool to thoroughly analyze the potential re-equilibration of brain metabolism that might be caused by metabolic mutations or to test new hypotheses based on new experimental data. With our model, we were able to provide novel insights about the pathophysiology and treatment of GLUT1-DS, including reassessing KD mechanisms, the role of astrocytes in metabolic buffering, seizure mechanisms, as well as the potential effects of supplementary treatments involving redox restoration and glucose-sparing. While acknowledging their limitations, computational merit consideration in informing future experiments and treatments. The study underscores the new model’s accuracy in replicating known biological processes and contributes to the identification of promising therapeutic avenues. Further experimental validation is necessary to confirm these predictions.

### Assumptions and limitations

As with any computational model, our GLUT1-DS model is subject to similar limitations regarding the availability of adequate data with which we build and validate the model, as well as biological realism. Implicit in our results interpretation are assumptions that the way we built the model, including its computational short-cuts, anatomical reductions and incomplete metabolic networks, allow us to nonetheless make insightful observations and predictions, upon which further focused research can be done. A nearly universal problem with biomolecular network models is the lack of spatial resolution in data coming from experimental laboratories. For such large metabolic networks, we don’t actually have much knowledge about whether metabolites are well mixed within cells or not, especially considering the complex geometries of brain cells (e.g., [91]).

In addition, we need to be careful about which questions to ask of the model, making sure the model is actually capable of providing a cogent answer within capabilities. For these reasons we are careful not to overreach in our interpretations. With regard to GLUT1-DS, there are many additional simulations that remain to be done, such as examining the effects of damage to astrocytic endfeet [92], the secondary, non-energy functions of lactate [93], more comprehensive lipid or ketone body metabolism [94] and the other pathways of brain energy rescue previously outlined [6]. The dynamics of the vicious cycle of energy deficiency in neurodegeneration are also missing from the current model but are likely to also play a role in GLUT1-DS just as they do in other disorders [6]. In addition, it should be noted that interpatient variability and long-term disease state changes are not assessed in this iteration of the model, although they could be, given the availability of appropriate data.

## Materials and methods

Our study is entirely computational and employs the most detailed and biologically accurate model of the NGV currently in existence [55] (Shichkova et al., 2025). Although built partially on previous models [39,95–98] (Berndt et al., 2015; Jolivet et al., 2015; Winter et al., 2018; Theurey et al., 2019; Coggan et al., 2022), this new model is dramatically improved, with **151** differential equations, **183** processes (**95** enzymatic reactions, **19** processes of transport of molecules across the cell and mitochondrial membranes, and **69** other processes related to ionic currents, blood flow dynamics and some miscellaneous non-enzymatic processes), **682** parameters and **151** variables describing metabolic reactions presenting glycolysis, TCA, OXPHOS, glycogenolysis and transporters distributed across a neuron, astrocyte, epithelia and the local vasculature (for blood-borne nutrients).

### Model

To better account for the mechanisms that are potentially involved in GLUT1-deficiency syndrome, we extended our earlier NGV model [55] (Shichkova et al., 2025) by adding glucose concentration dependence to Na^+^/K^+^ ATPase in the neuron and pyruvate carboxylase (PC) in the neuron. This also permitted investigation of the effect of GLUT1-deficiency on the excitation/inhibition balance as suggested recently [29] (Rajasekaran et al., 2022). While our original NGV model [55] (Shichkova et al., 2025) had only an astrocytic PC, here we also modeled it in the neuron– but at a lower expression level than in astrocyte. This has been done to avoid potential bias between two cells while testing pyruvate-related therapies. For the ATP titration to treat the seizure phenotype as shown in Fig 5, The 5 incremental doses of exogenous ATP correspond to 5 separate simulations, one per each dose. The application of exogenous ATP is reflected by initial values for cytosolic and mitochondrial IMS ATP concentrations, as well as the total cytosolic adenosine pool.

The model is implemented and simulated in the Julia scientific computing language [99] (Bezanson et al., 2014) and relied on use of the DifferentialEquations.jl package. Numerical solver parameters are set the same as for our earlier model [55] (Shichkova et al., 2025). Figures are generated using standard Python libraries such as seaborn and matplotlib.

### GLUT1-deficiency syndrome implementation

GLUT1-DS is characterized by changes in the kinetics of glucose transporters [100] (Wang et al., 2008) and associated changes in the ratio of cerebrospinal fluid (CSF)/blood glucose [101,102] (Leturque et al., 2012; Tan et al., 2023), concentration of CSF lactate [31] (Klepper et al., 2020), and total brain glycogen [29] (Rajasekaran et al., 2022). The multiplication coefficient change for most parameters in GLUT1-DS is given as a ratio or percent change from healthy controls in the literature (Table A in S1 Text). These coefficients are based on the references in the right column of the table. Kinetic parameters are taken directly from the text concentrations, but subject to scaling. Scaling for kinetic parameters is introduced according to how they influence concentrations [100] (e.g., Wang et al. 2008). In addition, all the used to construct the figures in this paper are provided in the Supplemental Material (Table B in S1 Text).

## Supporting information

S1 Text Supplemental figures, legends and tables. (ZIP)

S1 Data The GLUT1-DS model files. (ZIP)
