# Supplementary material for: Seizure and redox rescue in a model of glucose transport deficiency
Source: PLoS Comput Biol. 2025 Apr 4;21(4):e1012959. doi: 10.1371/journal.pcbi.1012959 (PMC12002639; doi:10.1371/journal.pcbi.1012959)

## **S1 Text**

## **Supplemental figures, legends and tables.**

**Fig A in S1 Text**


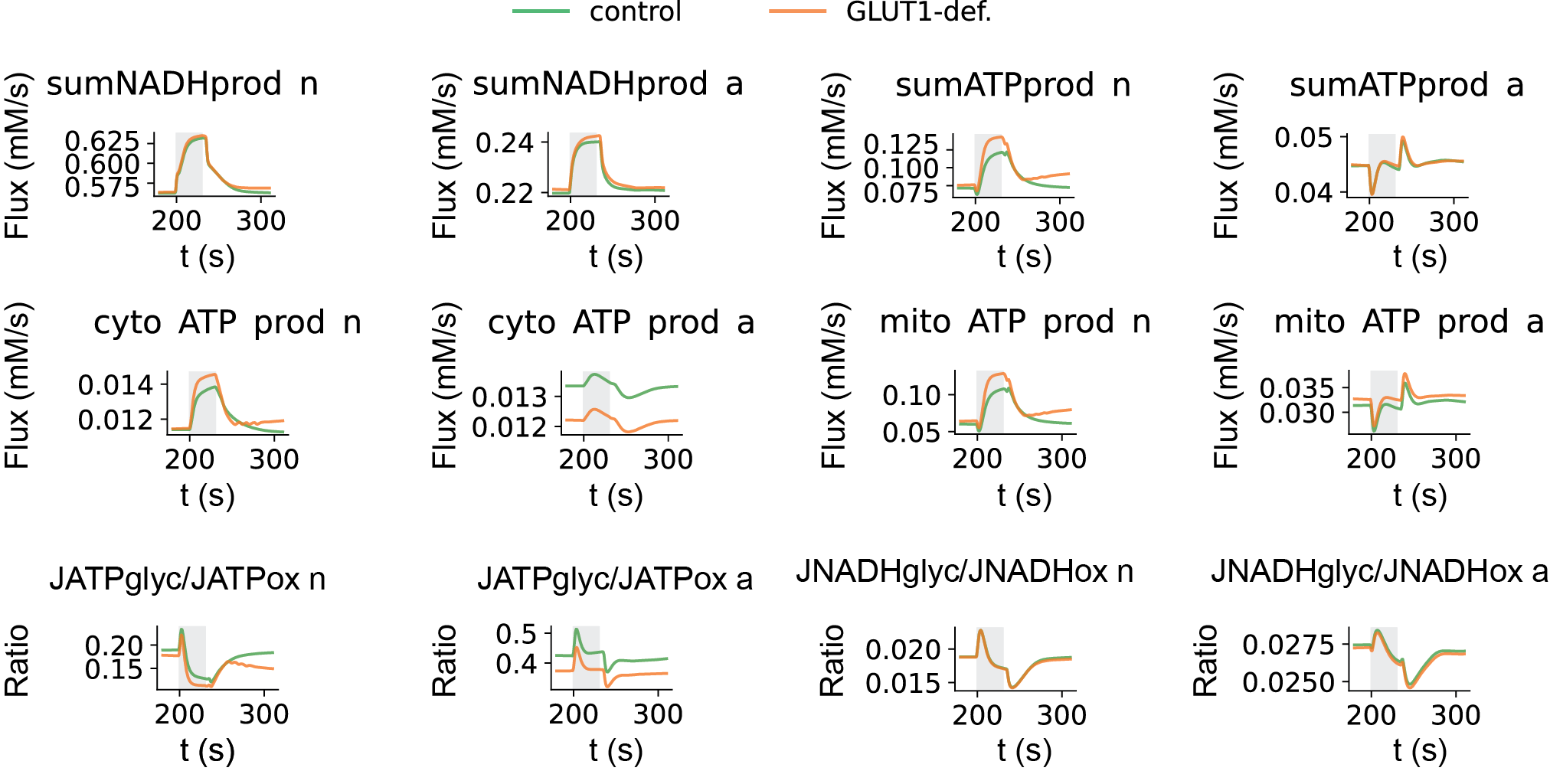


**Figure A in S1 Text**. Selected fluxes for redox and energy relevant metabolites (ATP and NADH) in neurons (n) and astrocytes (a), as well as ratios of some selected fluxes (J…/J…) in the bottom row.

**Fig B in S1 Text**

**
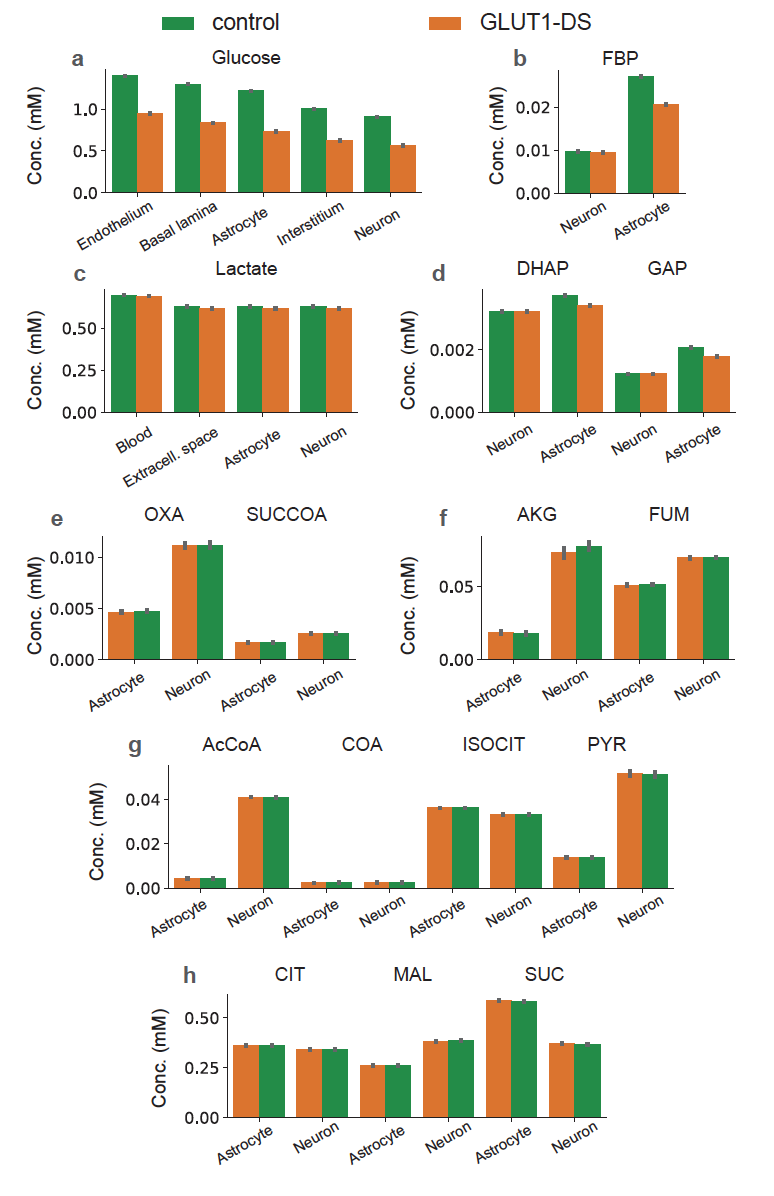
**

**Figure B in S1 Text**. The fate of selected energy metabolites with relation to specific cellular functions in GLUT1-DS. a) the significant reduction of GLC in various compartments, GLC in endothelium, extracellular space from blood to astrocyte, GLC in the astrocyte, GLC in the extracellular space between astrocyte and neuron, or interstitium, GLC in the neuron (the data in this panel is repeated from figure 1c in the main text for convenient comparison to related metabolites). b) fructose bisphosphatase (FBP), change only in the astrocyte), results correspond to the increased sensitivity of FBP in the astrocyte’s preference for glycolytic upregulation. c) LAC not significantly affected by GLUT1-DS, blood LAC, extracellular LAC, astrocyte LAC, neuron LAC. d) dehydroxyacetone phosphate (DHAP) and glyceraldehyde-3-phosphate (GAP). DHAP and GAP only reduced in astrocyte and are also important for the astrocytic preference for glycolysis as the points of regulation involving methylglyoxal. e) no effects of GLUT1-DS on points of anaplerotic regulation, oxaloacetate (OXA), succinylCoA (SUCCOA), a-ketoglutarate (AKG), and fumarate (FUM), f) other TCA metabolites not affected in either neuron or astrocyte, acetylCoA (AcCoA), coenzymeA (CoA), isocitrate (ISOCIT), pyruvate (PYR), citrate (CIT), malate (MAL), succinate (SUC).

**Fig C in S1 Text**


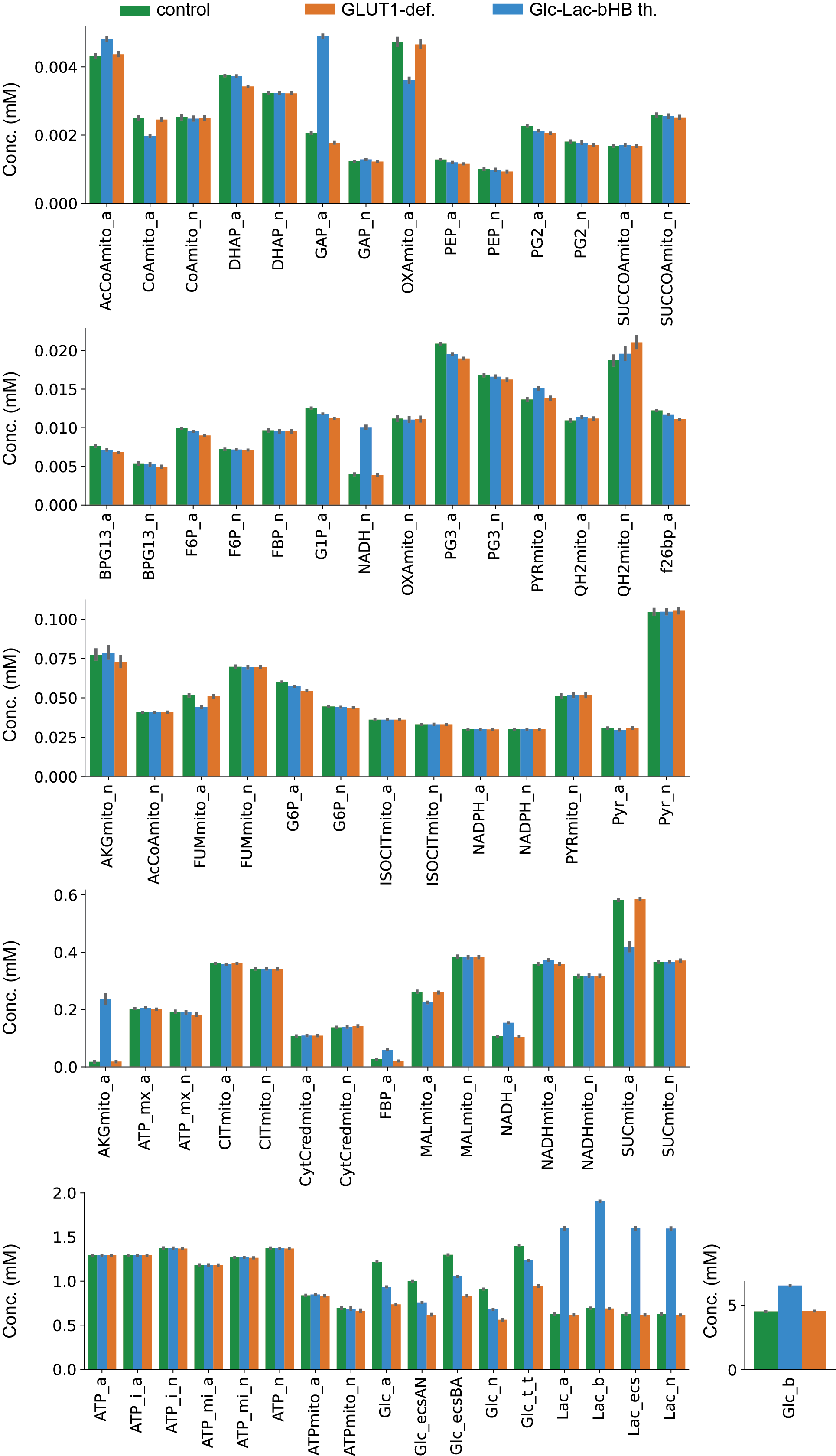


**Figure C in S1 Text**. The responses of energy metabolites in the cytoplasm and mitochondria for neurons and astrocytes in control, GLUT1-def and the GLC-LAC-bHB highest rank therapy for redox recovery. In neurons (_n) and astrocytes (_a).

**Figure D in S1 Text**


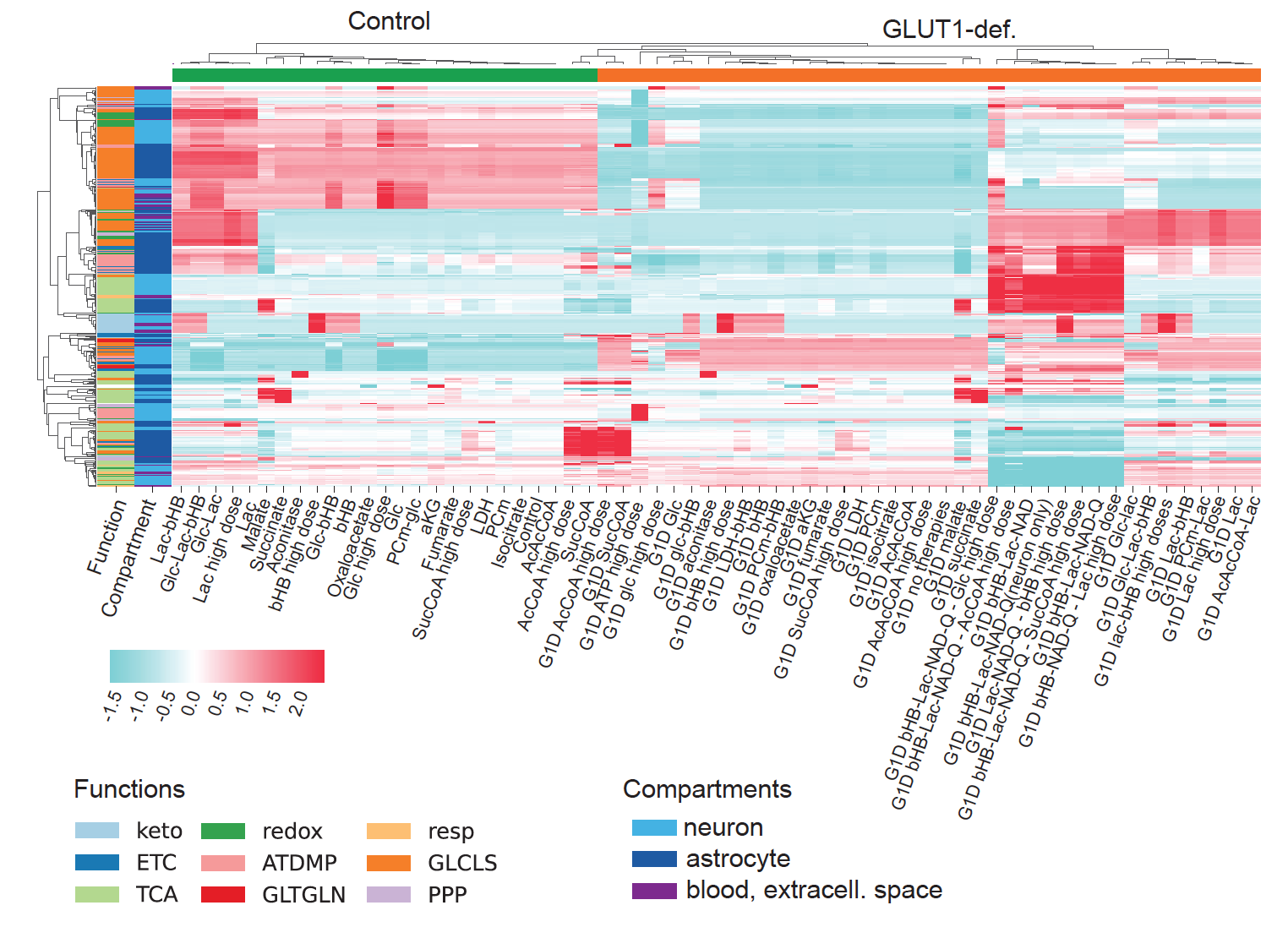


**Figure D in S1 Text**. Heat map implemented with average clustering with cosine metric comparing relative changes in metabolite levels in control and GLUT1-DS in multiple compartments (neuron, astrocyte, blood, extracellular space), and clustered by metabolic function: ketogenic diet (keto), redox metabolites (redox), respiration excluding electron transport chain (resp), electron transport chain (ETC), adenosine phosphates (ATDMP), glycolysis (GLCLS), TCA cycle (TCA), glutamate-glutamine cycle (GLTGLN), pentose phosphate shunt (PPP). Figure was generated in Python using the clustermap function of the seaborn package.

**Table A in S1 Text**

**Table A in S1 Text: GLUT1-deficiency syndrome factors implemented as input to the model.**

The multiplication coefficient for relevant parameters in GLUT1-DS is given as a ratio or percent change from healthy controls from previous reports (Supplemental Table 1). These coefficients are derived from the references in the right column. Some kinetic parameters were subject to scaling according to impact on concentrations. For example, from Tan et al (2023), for efflux and influx conditions, the T295M Vmax (590 pmol/min/oocyte) was 79% of the WT value and the Km (14.3 mM) was increased compared with WT (9.6 mM). For influx and efflux, both the Vmax (1216 pmol/min/oocyte) and Km (8.8 mM) were decreased in comparison to the WT values (7443 pmol/min/oocyte and 90.8 mM).

| Feature | Value, how it is derived | Comment | References |
| --- | --- | --- | --- |
| Kinetics of glucose transporters | # blood -> endothelial # influx  TmaxGLCce = 0.79*2.21  # endothelial -> ecsBA # efflux  TmaxGLCeb = 0.16*20.0  # ecsBA -> a # influx  TmaxGLCba = 0.79*8.0  # a -> ecsAN # efflux  TmaxGLCai = 0.16*0.032  KeG = (14.3/9.6)*10.3 # influx  KeG2 = (8.8/90.8)*12.5 # efflux  KeG3 = (14.3/9.6)*8.0 # influx  KeG4 = (8.8/90.8)*8.0 # efflux | Efflux is affected, but influx is almost unaffected. Detailed compartmentalization. | Wang et al., 2008 [<https://www.nature.com/articles/pr2008239>] |
| Basal lamina glucose (u0_ss[117] == Glc_ecsBA) | 0.69*1.302331013714969 | Efflux from endothelium is affected, so that basal lamina glucose concentration is affected accordingly (0.45/0.65 ~ 0.69) | Wang et al., (2008) [<https://www.nature.com/articles/pr2008239>]  Simmons, RA (2017)  [<https://www.sciencedirect.com/topics/biochemistry-genetics-and-molecular-biology/glut1#:~:text=GLUT1%2DDS%20is%20diagnosed%20when,feeding%20patients%20a%20ketogenic%20diet>.] (“The cerebrospinal fluid glucose/blood glucose concentration ratios in GLUT1-DS patients are about 0.45 (normal ratio: 0.65 ± 0.01)”)] |
| Astrocytic glucose (u0_ss[118] == Glc_a) | 0.69*1.2215129412034005 | Import is almost unaffected, but basal lamina glucose is affected by export from endothelium, so available for import pool of glucose is reduced | Wang et al., (2008) [<https://www.nature.com/articles/pr2008239>];  Simmons, RA (2017)  [<https://www.sciencedirect.com/topics/biochemistry-genetics-and-molecular-biology/glut1#:~:text=GLUT1%2DDS%20is%20diagnosed%20when,feeding%20patients%20a%20ketogenic%20diet>]. (“The cerebrospinal fluid glucose/blood glucose concentration ratios in GLUT1-DS patients are about 0.45 (normal ratio: 0.65 ± 0.01)”)] |
| Interstitial space glucose (u0_ss[119] == Glc_ecsAN) | 0.69*0.69*1.0034276408975709 | Both exports from endothelium and astrocyte are affected. | Wang et al., (2008) [<https://www.nature.com/articles/pr2008239>]  Simmons, RA (2017)  [<https://www.sciencedirect.com/topics/biochemistry-genetics-and-molecular-biology/glut1#:~:text=GLUT1%2DDS%20is%20diagnosed%20when,feeding%20patients%20a%20ketogenic%20diet>]. (“The cerebrospinal fluid glucose/blood glucose concentration ratios in GLUT1-DS patients are about 0.45 (normal ratio: 0.65 ± 0.01)”)] |
| Extracellular space lactate (u0_ss[150] == Lac_ecs) | 0.9*0.6223377821910432 | Slightly lowered due to low to low-normal CSF lactate | Klepper et al., (2020) [<https://www.ncbi.nlm.nih.gov/pmc/articles/PMC7469861/> ] |
| Astrocytic glycogen (u0_ss[128]) | (0.65/1.44)*13.99969995417289 |  | Rajasekaran et al., (2022) [DOI:10.1126/scitranslmed.abn2956] |

**Therapies implementation**

**Supplementary Table 2: Potential GLUT1-deficiency syndrome therapies tested in the model.**

| Therapy id | Description (only therapy-related parameters are listed, GLUT1-DS parameters are given in Table 1 and are also applied for all therapies). |
| --- | --- |
| g1_21_blood_glc_ini | Slightly increased blood glucose.  C_Glc_a = 6.6 mM (arterial glucose)  u0_ss[115] = (C_Glc_a/4.6)*4.555436497117541  (capillary glucose initial value) |
| g1_22_blood_lac_ini | Slightly increased blood lactate.  C_Lac_a = 2.0 mM (arterial lactate)  u0_ss[149] = (C_Lac_a/0.75)*0.6887631395085969 (capillary lactate initial value) |
| g1_23_blood_bhb_ini | Increased blood, astrocyte and extracellular β-hydroxybutyrate.  C_bHB_a = 3.3 mM (arterial bHB)  u0_ss[42] = (C_bHB_a/0.3)*0.2981203841045341 (capillary bHB initial value)  u0_ss[41] = (C_bHB_a/0.3)*0.002235 (astrocyte bHB, fixed)  u0_ss[40] = (C_bHB_a/0.3)*0.0028617229372728 (extracellular bHB) |
| g1_24_aKG_ini | Increased a-ketoglutarate  u0_ss[31] = 3.0*0.0730279198447203 (AKGmito_n initial value)  u0_ss[48] = 3.0*0.2 (AKG_n initial value)  u0_ss[81] = 3.0*0.0151591579926322 (AKGmito_a initial value) |
| g1_25_succoa_ini | Increased SucCoA  u0_ss[29] = 3.0*0.0027652024434962 (initial value)  u0_ss[79] = 3.0*0.0016699868314113 (initial value) |
| g1_26_fum_ini | Increased fumarate  u0_ss[25] = 3.0*0.0703303834778912 (initial value)  u0_ss[75] = 3.0*0.0500591772559637 (initial value) |
| g1_27_mal_ini | Increased malate  u0_ss[26] = 3.0*0.3877284278881228 (initial value)  u0_ss[46] = 3.0*0.45 (initial value)  u0_ss[76] = 3.0*0.2552499695609829 (initial value) |
| g1_28_oxa_ini | Increased oxaloacetate  u0_ss[27] = 3.0*0.0113308326310003  u0_ss[47] = 3.0*0.01  u0_ss[77] = 3.0*0.0044682268709836 |
| g1_37_glclac_ini | Slightly increased blood glucose and lactate  C_Glc_a = 6.6 mM (arterial glucose)  u0_ss[115] = (C_Glc_a/4.6)*4.555436497117541  (capillary glucose initial value)  C_Lac_a = 2.0 mM (arterial lactate)  u0_ss[149] = (C_Lac_a/0.75)*0.6887631395085969 (capillary lactate initial value) |
| g1_38_glcbhb_ini | Slightly increased blood glucose and bHB (blood, astrocyte, extracellular)  C_Glc_a = 6.6 mM (arterial glucose)  u0_ss[115] = (C_Glc_a/4.6)*4.555436497117541  (capillary glucose initial value)  C_bHB_a = 3.3 mM (arterial bHB)  u0_ss[42] = (C_bHB_a/0.3)*0.2981203841045341 (capillary bHB initial value)  u0_ss[41] = (C_bHB_a/0.3)*0.002235 (astrocyte bHB, fixed)  u0_ss[40] = (C_bHB_a/0.3)*0.0028617229372728 (extracellular bHB) |
| g1_39_lacbhb_ini | Slightly increased blood lactate and bHB (blood, astrocyte, extracellular)  C_Lac_a = 2.0 mM (arterial lactate)  u0_ss[149] = (C_Lac_a/0.75)*0.6887631395085969 (capillary lactate initial value)  C_bHB_a = 3.3 mM (arterial bHB)  u0_ss[42] = (C_bHB_a/0.3)*0.2981203841045341 (capillary bHB initial value)  u0_ss[41] = (C_bHB_a/0.3)*0.002235 (astrocyte bHB, fixed)  u0_ss[40] = (C_bHB_a/0.3)*0.0028617229372728 (extracellular bHB) |
| g1_40_glclacbhb_ini | Slightly increased blood glucose, lactate and bHB (blood, astrocyte, extracellular)  C_Glc_a = 6.6 mM (arterial glucose)  u0_ss[115] = (C_Glc_a/4.6)*4.555436497117541  (capillary glucose initial value)  C_Lac_a = 2.0 mM (arterial lactate)  u0_ss[149] = (C_Lac_a/0.75)*0.6887631395085969 (capillary lactate initial value)  C_bHB_a = 3.3 mM (arterial bHB)  u0_ss[42] = (C_bHB_a/0.3)*0.2981203841045341 (capillary bHB initial value)  u0_ss[41] = (C_bHB_a/0.3)*0.002235 (astrocyte bHB, fixed)  u0_ss[40] = (C_bHB_a/0.3)*0.0028617229372728 (extracellular bHB) |
| g1_65_PCm_keto | Increased Vmax of PCm in both neuron and astrocyte, increased bHB (blood, astrocyte, extracellular)  VmPYRCARB = 3.0*0.00755985436706299 - this change affects both n and a, because the parameter is shared between two cell types, additional scaling factor is involved in rate equation of neuronal PCm to reflect cell-type dependent difference in expression  C_bHB_a = 3.3 mM (arterial bHB)  u0_ss[42] = (C_bHB_a/0.3)*0.2981203841045341 (capillary bHB initial value)  u0_ss[41] = (C_bHB_a/0.3)*0.002235 (astrocyte bHB, fixed)  u0_ss[40] = (C_bHB_a/0.3)*0.0028617229372728 (extracellular bHB) |
| g1_66_PCm_lac | Increased Vmax of PCm in both neuron and astrocyte, increased lactate  VmPYRCARB = 3.0*0.00755985436706299 - this change affects both n and a, because the parameter is shared between two cell types, additional scaling factor is involved in rate equation of neuronal PCm to reflect cell-type dependent difference in expression  C_Lac_a = 2.0 mM (arterial lactate)  u0_ss[149] = (C_Lac_a/0.75)*0.6887631395085969 (capillary lactate initial value) |
| g1_67_PCm | Increased Vmax of PCm in both neuron and astrocyte  VmPYRCARB = 3.0*0.00755985436706299 - this change affects both n and a, because the parameter is shared between two cell types, additional scaling factor is involved in rate equation of neuronal PCm to reflect cell-type dependent difference in expression |
| g1_68_AcAcCoA | Increased initial concentrations of acetoacetyl-CoA in neuron and astrocyte  u0_ss[37] = 3.0*3.471725572540322e-5 (AcAcCoA in neuron)  u0_ss[87] = 3.0*0.0006 (AcAcCoA_a in astrocyte) |
| g1_69_Acon | Reduced Vmax of aconitase in neuron and astrocyte  VmaxAco_n = 0.5*25.611147830094392  VmaxAco_a = 0.5*9.438075110105698 |
| g1_70_ISOCIT | Increased initial concentration of isocitrate in neuron and astrocyte  u0_ss[33] = 3.0*0.0331278125743512  u0_ss[83] = 3.0*0.0360692382798456 |
| g1_71_LDH_keto | Reduced Vmax of lactate dehydrogenase in neuron and astrocyte and increased concentration of bHB (blood, astrocyte, extracellular)  VmfLDH_a = 0.5*8.74949881831907  VmfLDH_n = 0.5*241.739831262545  C_bHB_a = 3.3 mM (arterial bHB)  u0_ss[42] = (C_bHB_a/0.3)*0.2981203841045341 (capillary bHB initial value)  u0_ss[41] = (C_bHB_a/0.3)*0.002235 (astrocyte bHB, fixed)  u0_ss[40] = (C_bHB_a/0.3)*0.0028617229372728 (extracellular bHB) |
| g1_72_LDH | Reduced Vmax of lactate dehydrogenase in neuron and astrocyte  VmfLDH_a = 0.5*8.74949881831907  VmfLDH_n = 0.5*241.739831262545 |
| g1_73_PDH_keto | Reduced Vmax of pyruvate dehydrogenase and increased concentration of bHB (blood, astrocyte, extracellular)  VmaxPDHCmito_n = 0.5*2.67222326259307  VmaxPDHCmito_a = 0.5*2.79810789599674  C_bHB_a = 3.3 mM (arterial bHB)  u0_ss[42] = (C_bHB_a/0.3)*0.2981203841045341 (capillary bHB initial value)  u0_ss[41] = (C_bHB_a/0.3)*0.002235 (astrocyte bHB, fixed)  u0_ss[40] = (C_bHB_a/0.3)*0.0028617229372728 (extracellular bHB) |
| g1_74_PDH_lac | Reduced Vmax of pyruvate dehydrogenase and increased concentration of blood lactate  VmaxPDHCmito_n = 0.5*2.67222326259307  VmaxPDHCmito_a = 0.5*2.79810789599674  C_Lac_a = 2.0 mM (arterial lactate)  u0_ss[149] = (C_Lac_a/0.75)*0.6887631395085969 (capillary lactate initial value) |
| g1_75_PDH | Reduced Vmax of pyruvate dehydrogenase  VmaxPDHCmito_n = 0.5*2.67222326259307  VmaxPDHCmito_a = 0.5*2.79810789599674 |
| g1_76_SUCmito | Increased initial concentration of succinate in neuron and astrocyte  u0_ss[28] = 3.0*0.3913984292428137  u0_ss[78] = 3.0*0.5865999694835419 |
| g1_90_AcAcCoa_lac | Increased concentration of acetoacetyl-CoA (neuron, astrocyte) and blood lactate  u0_ss[37] = 3.0*3.471725572540322e-5 (AcAcCoA_n)  u0_ss[87] = 3.0*0.0006 (AcAcCoA_a)  C_Lac_a = 2.0 mM (arterial lactate)  u0_ss[149] = (C_Lac_a/0.75)*0.6887631395085969 (capillary lactate initial value) |
| g1_91_PDH_Lac_keto | Reduced Vmax of pyruvate dehydrogenase, increased initial concentration of blood lactate and bHB (blood, astrocyte, extracellular)  VmaxPDHCmito_n = 0.5*2.67222326259307  VmaxPDHCmito_a = 0.5*2.79810789599674  C_Lac_a = 2.0 mM (arterial lactate)  u0_ss[149] = (C_Lac_a/0.75)*0.6887631395085969 (capillary lactate initial value)  C_bHB_a = 3.3 mM (arterial bHB)  u0_ss[42] = (C_bHB_a/0.3)*0.2981203841045341 (capillary bHB initial value)  u0_ss[41] = (C_bHB_a/0.3)*0.002235 (astrocyte bHB, fixed)  u0_ss[40] = (C_bHB_a/0.3)*0.0028617229372728 (extracellular bHB) |
| g1_92_blood_glc_ini_dose1 | Reduced blood glucose.  C_Glc_a = 3.6 mM (arterial glucose)  u0_ss[115] = (C_Glc_a/4.6)*4.555436497117541  (capillary glucose initial value) |
| g1_93_blood_glc_ini_dose2 | Slightly increased blood glucose.  C_Glc_a = 5.6 mM (arterial glucose)  u0_ss[115] = (C_Glc_a/4.6)*4.555436497117541  (capillary glucose initial value) |
| g1_94_blood_glc_ini_dose3 | Slightly increased blood glucose.  C_Glc_a = 7.6 mM (arterial glucose)  u0_ss[115] = (C_Glc_a/4.6)*4.555436497117541  (capillary glucose initial value) |
| g1_95_blood_glc_ini_dose4 | Increased blood glucose.  C_Glc_a = 9.6 mM (arterial glucose)  u0_ss[115] = (C_Glc_a/4.6)*4.555436497117541  (capillary glucose initial value) |
| g1_96_blood_glc_ini_dose5 | Increased blood glucose.  C_Glc_a = 11.6 mM (arterial glucose)  u0_ss[115] = (C_Glc_a/4.6)*4.555436497117541  (capillary glucose initial value) |
| g1_97_blood_lac_ini_dose1 | Reduced blood lactate  C_Lac_a = 0.5 mM (arterial lactate)  u0_ss[149] = (C_Lac_a/0.75)*0.6887631395085969 (capillary lactate initial value) |
| g1_98_blood_lac_ini_dose2 | Slightly increased blood lactate  C_Lac_a = 1.0 mM (arterial lactate)  u0_ss[149] = (C_Lac_a/0.75)*0.6887631395085969 (capillary lactate initial value) |
| g1_99_blood_lac_ini_dose3 | Increased blood lactate  C_Lac_a = 1.5 mM (arterial lactate)  u0_ss[149] = (C_Lac_a/0.75)*0.6887631395085969 (capillary lactate initial value) |
| g1_100_blood_lac_ini_dose4 | Increased blood lactate  C_Lac_a = 1.75 mM (arterial lactate)  u0_ss[149] = (C_Lac_a/0.75)*0.6887631395085969 (capillary lactate initial value) |
| g1_101_blood_lac_ini_dose5 | Increased blood lactate  C_Lac_a = 2.25 mM (arterial lactate)  u0_ss[149] = (C_Lac_a/0.75)*0.6887631395085969 (capillary lactate initial value) |
| g1_102_blood_bhb_ini_dose1 | Increased blood, astrocyte and extracellular β-hydroxybutyrate.  C_bHB_a = 0.5 mM (arterial bHB)  u0_ss[42] = (C_bHB_a/0.3)*0.2981203841045341 (capillary bHB initial value)  u0_ss[41] = (C_bHB_a/0.3)*0.002235 (astrocyte bHB, fixed)  u0_ss[40] = (C_bHB_a/0.3)*0.0028617229372728 (extracellular bHB) |
| g1_103_blood_bhb_ini_dose2 | Increased blood, astrocyte and extracellular β-hydroxybutyrate.  C_bHB_a = 1.0 mM (arterial bHB)  u0_ss[42] = (C_bHB_a/0.3)*0.2981203841045341 (capillary bHB initial value)  u0_ss[41] = (C_bHB_a/0.3)*0.002235 (astrocyte bHB, fixed)  u0_ss[40] = (C_bHB_a/0.3)*0.0028617229372728 (extracellular bHB) |
| g1_104_blood_bhb_ini_dose3 | Increased blood, astrocyte and extracellular β-hydroxybutyrate.  C_bHB_a = 2.0 mM (arterial bHB)  u0_ss[42] = (C_bHB_a/0.3)*0.2981203841045341 (capillary bHB initial value)  u0_ss[41] = (C_bHB_a/0.3)*0.002235 (astrocyte bHB, fixed)  u0_ss[40] = (C_bHB_a/0.3)*0.0028617229372728 (extracellular bHB) |
| g1_105_blood_bhb_ini_dose4 | Increased blood, astrocyte and extracellular β-hydroxybutyrate.  C_bHB_a = 4.0 mM (arterial bHB)  u0_ss[42] = (C_bHB_a/0.3)*0.2981203841045341 (capillary bHB initial value)  u0_ss[41] = (C_bHB_a/0.3)*0.002235 (astrocyte bHB, fixed)  u0_ss[40] = (C_bHB_a/0.3)*0.0028617229372728 (extracellular bHB) |
| g1_106_blood_bhb_ini_dose5 | Increased blood, astrocyte and extracellular β-hydroxybutyrate.  C_bHB_a = 8.0 mM (arterial bHB)  u0_ss[42] = (C_bHB_a/0.3)*0.2981203841045341 (capillary bHB initial value)  u0_ss[41] = (C_bHB_a/0.3)*0.002235 (astrocyte bHB, fixed)  u0_ss[40] = (C_bHB_a/0.3)*0.0028617229372728 (extracellular bHB) |
| g1_107_blood_lacbhb_ini_dose1 | Reduced blood lactate, increased blood, astrocyte and extracellular β-hydroxybutyrate  C_Lac_a = 0.5 mM (arterial lactate)  u0_ss[149] = (C_Lac_a/0.75)*0.6887631395085969 (capillary lactate initial value)  C_bHB_a = 0.5 mM (arterial bHB)  u0_ss[42] = (C_bHB_a/0.3)*0.2981203841045341 (capillary bHB initial value)  u0_ss[41] = (C_bHB_a/0.3)*0.002235 (astrocyte bHB, fixed)  u0_ss[40] = (C_bHB_a/0.3)*0.0028617229372728 (extracellular bHB) |
| g1_108_blood_lacbhb_ini_dose2 | Slightly increased blood lactate, increased blood, astrocyte and extracellular β-hydroxybutyrate  C_Lac_a = 1.0 mM (arterial lactate)  u0_ss[149] = (C_Lac_a/0.75)*0.6887631395085969 (capillary lactate initial value)  C_bHB_a = 1.0 mM (arterial bHB)  u0_ss[42] = (C_bHB_a/0.3)*0.2981203841045341 (capillary bHB initial value)  u0_ss[41] = (C_bHB_a/0.3)*0.002235 (astrocyte bHB, fixed)  u0_ss[40] = (C_bHB_a/0.3)*0.0028617229372728 (extracellular bHB) |
| g1_109_blood_lacbhb_ini_dose3 | Increased blood lactate, increased blood, astrocyte and extracellular β-hydroxybutyrate  C_Lac_a = 1.5 mM (arterial lactate)  u0_ss[149] = (C_Lac_a/0.75)*0.6887631395085969 (capillary lactate initial value)  C_bHB_a = 2.0 mM (arterial bHB)  u0_ss[42] = (C_bHB_a/0.3)*0.2981203841045341 (capillary bHB initial value)  u0_ss[41] = (C_bHB_a/0.3)*0.002235 (astrocyte bHB, fixed)  u0_ss[40] = (C_bHB_a/0.3)*0.0028617229372728 (extracellular bHB) |
| g1_110_blood_lacbhb_ini_dose4 | Increased blood lactate, increased blood, astrocyte and extracellular β-hydroxybutyrate  C_Lac_a = 1.75 mM (arterial lactate)  u0_ss[149] = (C_Lac_a/0.75)*0.6887631395085969 (capillary lactate initial value)  C_bHB_a = 4.0 mM (arterial bHB)  u0_ss[42] = (C_bHB_a/0.3)*0.2981203841045341 (capillary bHB initial value)  u0_ss[41] = (C_bHB_a/0.3)*0.002235 (astrocyte bHB, fixed)  u0_ss[40] = (C_bHB_a/0.3)*0.0028617229372728 (extracellular bHB) |
| g1_111_blood_lacbhb_ini_dose5 | Increased blood lactate, increased blood, astrocyte and extracellular β-hydroxybutyrate  C_Lac_a = 2.25 mM (arterial lactate)  u0_ss[149] = (C_Lac_a/0.75)*0.6887631395085969 (capillary lactate initial value)  C_bHB_a = 8.0 mM (arterial bHB)  u0_ss[42] = (C_bHB_a/0.3)*0.2981203841045341 (capillary bHB initial value)  u0_ss[41] = (C_bHB_a/0.3)*0.002235 (astrocyte bHB, fixed)  u0_ss[40] = (C_bHB_a/0.3)*0.0028617229372728 (extracellular bHB) |
| g1_112_blood_lac_ini_dose6 | Increased blood lactate  C_Lac_a = 1.125 mM (arterial lactate)  u0_ss[149] = (C_Lac_a/0.75)*0.6887631395085969 (capillary lactate initial value) |
| g1_113_blood_lac_ini_dose7 | Increased blood lactate  C_Lac_a = 1.25 mM (arterial lactate)  u0_ss[149] = (C_Lac_a/0.75)*0.6887631395085969 (capillary lactate initial value) |
| g1_114_blood_lac_ini_dose8 | Increased blood lactate  C_Lac_a = 2.125 mM (arterial lactate)  u0_ss[149] = (C_Lac_a/0.75)*0.6887631395085969 (capillary lactate initial value) |
| g1_115_blood_lacbhb_ini_dose6 | Increased blood lactate, increased blood, astrocyte and extracellular β-hydroxybutyrate  C_Lac_a = 1.125 mM (arterial lactate)  u0_ss[149] = (C_Lac_a/0.75)*0.6887631395085969 (capillary lactate initial value)  C_bHB_a = 2.0 mM (arterial bHB)  u0_ss[42] = (C_bHB_a/0.3)*0.2981203841045341 (capillary bHB initial value)  u0_ss[41] = (C_bHB_a/0.3)*0.002235 (astrocyte bHB, fixed)  u0_ss[40] = (C_bHB_a/0.3)*0.0028617229372728 (extracellular bHB) |
| g1_116_blood_lacbhb_ini_dose7 | Increased blood lactate, increased blood, astrocyte and extracellular β-hydroxybutyrate  C_Lac_a = 1.25 mM (arterial lactate)  u0_ss[149] = (C_Lac_a/0.75)*0.6887631395085969 (capillary lactate initial value)  C_bHB_a = 4.0 mM (arterial bHB)  u0_ss[42] = (C_bHB_a/0.3)*0.2981203841045341 (capillary bHB initial value)  u0_ss[41] = (C_bHB_a/0.3)*0.002235 (astrocyte bHB, fixed)  u0_ss[40] = (C_bHB_a/0.3)*0.0028617229372728 (extracellular bHB) |
| g1_117_blood_lacbhb_ini_dose8 | Increased blood lactate, increased blood, astrocyte and extracellular β-hydroxybutyrate  C_Lac_a = 2.125 mM (arterial lactate)  u0_ss[149] = (C_Lac_a/0.75)*0.6887631395085969 (capillary lactate initial value)  C_bHB_a = 8.0 mM (arterial bHB)  u0_ss[42] = (C_bHB_a/0.3)*0.2981203841045341 (capillary bHB initial value)  u0_ss[41] = (C_bHB_a/0.3)*0.002235 (astrocyte bHB, fixed)  u0_ss[40] = (C_bHB_a/0.3)*0.0028617229372728 (extracellular bHB) |
| g1_118_keto_lac_nad | Increased blood, astrocyte and extracellular β-hydroxybutyrate; slightly increased blood lactate; increased mito-NAD pool  C_bHB_a = 3.3 mM (arterial bHB)  u0_ss[42] = (C_bHB_a/0.3)*0.2981203841045341 (capillary bHB initial value)  u0_ss[41] = (C_bHB_a/0.3)*0.002235 (astrocyte bHB, fixed)  u0_ss[40] = (C_bHB_a/0.3)*0.0028617229372728 (extracellular bHB)  C_Lac_a = 2.0 mM (arterial lactate)  u0_ss[149] = (C_Lac_a/0.75)*0.6887631395085969 (capillary lactate initial value)  NADtot = 1.1*0.000726 (mito neuron, astrocyte) |
| g1_119_keto_lac_nad_Qtot_n | Increased blood, astrocyte and extracellular β-hydroxybutyrate; slightly increased blood lactate; increased mito-NAD pool; increased initial concentration of coenzyme Q in neuron  C_bHB_a = 3.3 mM (arterial bHB)  u0_ss[42] = (C_bHB_a/0.3)*0.2981203841045341 (capillary bHB initial value)  u0_ss[41] = (C_bHB_a/0.3)*0.002235 (astrocyte bHB, fixed)  u0_ss[40] = (C_bHB_a/0.3)*0.0028617229372728 (extracellular bHB)  C_Lac_a = 2.0 mM (arterial lactate)  u0_ss[149] = (C_Lac_a/0.75)*0.6887631395085969 (capillary lactate initial value)  NADtot = 1.1*0.000726 (mito neuron, astrocyte)  u0_ss[21] = 1.1*1.35 |
| g1_120_keto_lac_nad_Qtot_na | Increased blood, astrocyte and extracellular β-hydroxybutyrate; slightly increased blood lactate; increased mito-NAD pool; increased initial concentration of coenzyme Q in neuron and astrocyte  C_bHB_a = 3.3 mM (arterial bHB)  u0_ss[42] = (C_bHB_a/0.3)*0.2981203841045341 (capillary bHB initial value)  u0_ss[41] = (C_bHB_a/0.3)*0.002235 (astrocyte bHB, fixed)  u0_ss[40] = (C_bHB_a/0.3)*0.0028617229372728 (extracellular bHB)  C_Lac_a = 2.0 mM (arterial lactate)  u0_ss[149] = (C_Lac_a/0.75)*0.6887631395085969 (capillary lactate initial value)  NADtot = 1.1*0.000726 (mito neuron, astrocyte)  u0_ss[21] = 1.1*1.35  u0_ss[71] = 1.1*1.35 |
| g1_122_AcAcCoA_ini_dose1 | Increased AcAcCoA initial concentration in neuron and astrocyte  u0_ss[37] = 1.1*3.471725572540322e-5  u0_ss[87] = 1.1*0.0006 |
| g1_123_AcAcCoA_ini_dose2 | Increased AcAcCoA initial concentration in neuron and astrocyte  u0_ss[37] = 1.2*3.471725572540322e-5  u0_ss[87] = 1.2*0.0006 |
| g1_124_AcAcCoA_ini_dose3 | Increased AcAcCoA initial concentration in neuron and astrocyte  u0_ss[37] = 1.3*3.471725572540322e-5  u0_ss[87] = 1.3*0.0006 |
| g1_125_AcAcCoA_ini_dose4 | Increased AcAcCoA initial concentration in neuron and astrocyte  u0_ss[37] = 1.4*3.471725572540322e-5  u0_ss[87] = 1.4*0.0006 |
| g1_126_AcAcCoA_ini_dose5 | Increased AcAcCoA initial concentration in neuron and astrocyte  u0_ss[37] = 1.5*3.471725572540322e-5  u0_ss[87] = 1.5*0.0006 |
| g1_127_AcCoAmito_na_ini_dose1 | Increased AcCoA initial concentration in neuron and astrocyte  u0_ss[35] = 1.1*0.040486317761993  u0_ss[85] = 1.1*0.0042886443294129 |
| g1_128_AcCoAmito_na_ini_dose2 | Increased AcCoA initial concentration in neuron and astrocyte  u0_ss[35] = 1.2*0.040486317761993  u0_ss[85] = 1.2*0.0042886443294129 |
| g1_129_AcCoAmito_na_ini_dose3 | Increased AcCoA initial concentration in neuron and astrocyte  u0_ss[35] = 1.3*0.040486317761993  u0_ss[85] = 1.3*0.0042886443294129 |
| g1_130_AcCoAmito_na_ini_dose4 | Increased AcCoA initial concentration in neuron and astrocyte  u0_ss[35] = 1.4*0.040486317761993  u0_ss[85] = 1.4*0.0042886443294129 |
| g1_131_AcCoAmito_na_ini_dose5 | Increased AcCoA initial concentration in neuron and astrocyte  u0_ss[35] = 1.5*0.040486317761993  u0_ss[85] = 1.5*0.0042886443294129 |
| g1_132_SUCCOAmito_na_ini_dose1 | Increased succinate-CoA initial concentration in neuron and astrocyte  u0_ss[29] = 1.1*0.0027652024434962  u0_ss[79] = 1.1*0.0016699868314113 |
| g1_133_SUCCOAmito_na_ini_dose2 | Increased succinate-CoA initial concentration in neuron and astrocyte  u0_ss[29] = 1.2*0.0027652024434962  u0_ss[79] = 1.2*0.0016699868314113 |
| g1_134_SUCCOAmito_na_ini_dose3 | Increased succinate-CoA initial concentration in neuron and astrocyte  u0_ss[29] = 1.3*0.0027652024434962  u0_ss[79] = 1.3*0.0016699868314113 |
| g1_135_SUCCOAmito_na_ini_dose4 | Increased succinate-CoA initial concentration in neuron and astrocyte  u0_ss[29] = 1.4*0.0027652024434962  u0_ss[79] = 1.4*0.0016699868314113 |
| g1_136_SUCCOAmito_na_ini_dose5 | Increased succinate-CoA initial concentration in neuron and astrocyte  u0_ss[29] = 1.5*0.0027652024434962  u0_ss[79] = 1.5*0.0016699868314113 |
| th_162_blood_glc_ini_dose1 | Increased blood, astrocyte and extracellular β-hydroxybutyrate; slightly increased blood lactate; increased mito-NAD pool; increased initial concentration of coenzyme Q in neuron and astrocyte, reduced blood glucose.  C_bHB_a = 3.3 mM (arterial bHB)  u0_ss[42] = (C_bHB_a/0.3)*0.2981203841045341 (capillary bHB initial value)  u0_ss[41] = (C_bHB_a/0.3)*0.002235 (astrocyte bHB, fixed)  u0_ss[40] = (C_bHB_a/0.3)*0.0028617229372728 (extracellular bHB)  C_Lac_a = 2.0 mM (arterial lactate)  u0_ss[149] = (C_Lac_a/0.75)*0.6887631395085969 (capillary lactate initial value)  NADtot = 1.1*0.000726 (mito neuron, astrocyte)  u0_ss[21] = 1.1*1.35  u0_ss[71] = 1.1*1.35  C_Glc_a = 3.6 mM (arterial glucose)  u0_ss[115] = (C_Glc_a/4.6)*4.555436497117541  (capillary glucose initial value) |
| th_163_blood_glc_ini_dose2 | Increased blood, astrocyte and extracellular β-hydroxybutyrate; slightly increased blood lactate; increased mito-NAD pool; increased initial concentration of coenzyme Q in neuron and astrocyte, slightly increased blood glucose.  C_bHB_a = 3.3 mM (arterial bHB)  u0_ss[42] = (C_bHB_a/0.3)*0.2981203841045341 (capillary bHB initial value)  u0_ss[41] = (C_bHB_a/0.3)*0.002235 (astrocyte bHB, fixed)  u0_ss[40] = (C_bHB_a/0.3)*0.0028617229372728 (extracellular bHB)  C_Lac_a = 2.0 mM (arterial lactate)  u0_ss[149] = (C_Lac_a/0.75)*0.6887631395085969 (capillary lactate initial value)  NADtot = 1.1*0.000726 (mito neuron, astrocyte)  u0_ss[21] = 1.1*1.35  u0_ss[71] = 1.1*1.35  C_Glc_a = 5.6 mM (arterial glucose)  u0_ss[115] = (C_Glc_a/4.6)*4.555436497117541  (capillary glucose initial value) |
| th_164_blood_glc_ini_dose3 | Increased blood, astrocyte and extracellular β-hydroxybutyrate; slightly increased blood lactate; increased mito-NAD pool; increased initial concentration of coenzyme Q in neuron and astrocyte, increased blood glucose.  C_bHB_a = 3.3 mM (arterial bHB)  u0_ss[42] = (C_bHB_a/0.3)*0.2981203841045341 (capillary bHB initial value)  u0_ss[41] = (C_bHB_a/0.3)*0.002235 (astrocyte bHB, fixed)  u0_ss[40] = (C_bHB_a/0.3)*0.0028617229372728 (extracellular bHB)  C_Lac_a = 2.0 mM (arterial lactate)  u0_ss[149] = (C_Lac_a/0.75)*0.6887631395085969 (capillary lactate initial value)  NADtot = 1.1*0.000726 (mito neuron, astrocyte)  u0_ss[21] = 1.1*1.35  u0_ss[71] = 1.1*1.35  C_Glc_a = 7.6 mM (arterial glucose)  u0_ss[115] = (C_Glc_a/4.6)*4.555436497117541  (capillary glucose initial value) |
| th_165_blood_glc_ini_dose4 | Increased blood, astrocyte and extracellular β-hydroxybutyrate; slightly increased blood lactate; increased mito-NAD pool; increased initial concentration of coenzyme Q in neuron and astrocyte, increased blood glucose.  C_bHB_a = 3.3 mM (arterial bHB)  u0_ss[42] = (C_bHB_a/0.3)*0.2981203841045341 (capillary bHB initial value)  u0_ss[41] = (C_bHB_a/0.3)*0.002235 (astrocyte bHB, fixed)  u0_ss[40] = (C_bHB_a/0.3)*0.0028617229372728 (extracellular bHB)  C_Lac_a = 2.0 mM (arterial lactate)  u0_ss[149] = (C_Lac_a/0.75)*0.6887631395085969 (capillary lactate initial value)  NADtot = 1.1*0.000726 (mito neuron, astrocyte)  u0_ss[21] = 1.1*1.35  u0_ss[71] = 1.1*1.35  C_Glc_a = 9.6 mM (arterial glucose)  u0_ss[115] = (C_Glc_a/4.6)*4.555436497117541  (capillary glucose initial value) |
| th_166_blood_glc_ini_dose5 | Increased blood, astrocyte and extracellular β-hydroxybutyrate; slightly increased blood lactate; increased mito-NAD pool; increased initial concentration of coenzyme Q in neuron and astrocyte, increased blood glucose.  C_bHB_a = 3.3 mM (arterial bHB)  u0_ss[42] = (C_bHB_a/0.3)*0.2981203841045341 (capillary bHB initial value)  u0_ss[41] = (C_bHB_a/0.3)*0.002235 (astrocyte bHB, fixed)  u0_ss[40] = (C_bHB_a/0.3)*0.0028617229372728 (extracellular bHB)  C_Lac_a = 2.0 mM (arterial lactate)  u0_ss[149] = (C_Lac_a/0.75)*0.6887631395085969 (capillary lactate initial value)  NADtot = 1.1*0.000726 (mito neuron, astrocyte)  u0_ss[21] = 1.1*1.35  u0_ss[71] = 1.1*1.35  C_Glc_a = 11.6 mM (arterial glucose)  u0_ss[115] = (C_Glc_a/4.6)*4.555436497117541  (capillary glucose initial value) |
| th_167_blood_lac_ini_dose1 | Increased blood, astrocyte and extracellular β-hydroxybutyrate; reduced blood lactate; increased mito-NAD pool; increased initial concentration of coenzyme Q in neuron and astrocyte.  C_bHB_a = 3.3 mM (arterial bHB)  u0_ss[42] = (C_bHB_a/0.3)*0.2981203841045341 (capillary bHB initial value)  u0_ss[41] = (C_bHB_a/0.3)*0.002235 (astrocyte bHB, fixed)  u0_ss[40] = (C_bHB_a/0.3)*0.0028617229372728 (extracellular bHB)  C_Lac_a = 0.5 mM (arterial lactate)  u0_ss[149] = (C_Lac_a/0.75)*0.6887631395085969 (capillary lactate initial value)  NADtot = 1.1*0.000726 (mito neuron, astrocyte)  u0_ss[21] = 1.1*1.35  u0_ss[71] = 1.1*1.35 |
| th_168_blood_lac_ini_dose2 | Increased blood, astrocyte and extracellular β-hydroxybutyrate; slightly increased blood lactate; increased mito-NAD pool; increased initial concentration of coenzyme Q in neuron and astrocyte.  C_bHB_a = 3.3 mM (arterial bHB)  u0_ss[42] = (C_bHB_a/0.3)*0.2981203841045341 (capillary bHB initial value)  u0_ss[41] = (C_bHB_a/0.3)*0.002235 (astrocyte bHB, fixed)  u0_ss[40] = (C_bHB_a/0.3)*0.0028617229372728 (extracellular bHB)  C_Lac_a = 1.0 mM (arterial lactate)  u0_ss[149] = (C_Lac_a/0.75)*0.6887631395085969 (capillary lactate initial value)  NADtot = 1.1*0.000726 (mito neuron, astrocyte)  u0_ss[21] = 1.1*1.35  u0_ss[71] = 1.1*1.35 |
| th_169_blood_lac_ini_dose3 | Increased blood, astrocyte and extracellular β-hydroxybutyrate; increased blood lactate; increased mito-NAD pool; increased initial concentration of coenzyme Q in neuron and astrocyte.  C_bHB_a = 3.3 mM (arterial bHB)  u0_ss[42] = (C_bHB_a/0.3)*0.2981203841045341 (capillary bHB initial value)  u0_ss[41] = (C_bHB_a/0.3)*0.002235 (astrocyte bHB, fixed)  u0_ss[40] = (C_bHB_a/0.3)*0.0028617229372728 (extracellular bHB)  C_Lac_a = 1.5 mM (arterial lactate)  u0_ss[149] = (C_Lac_a/0.75)*0.6887631395085969 (capillary lactate initial value)  NADtot = 1.1*0.000726 (mito neuron, astrocyte)  u0_ss[21] = 1.1*1.35  u0_ss[71] = 1.1*1.35 |
| th_170_blood_lac_ini_dose4 | Increased blood, astrocyte and extracellular β-hydroxybutyrate; increased blood lactate; increased mito-NAD pool; increased initial concentration of coenzyme Q in neuron and astrocyte.  C_bHB_a = 3.3 mM (arterial bHB)  u0_ss[42] = (C_bHB_a/0.3)*0.2981203841045341 (capillary bHB initial value)  u0_ss[41] = (C_bHB_a/0.3)*0.002235 (astrocyte bHB, fixed)  u0_ss[40] = (C_bHB_a/0.3)*0.0028617229372728 (extracellular bHB)  C_Lac_a = 1.75 mM (arterial lactate)  u0_ss[149] = (C_Lac_a/0.75)*0.6887631395085969 (capillary lactate initial value)  NADtot = 1.1*0.000726 (mito neuron, astrocyte)  u0_ss[21] = 1.1*1.35  u0_ss[71] = 1.1*1.35 |
| th_171_blood_lac_ini_dose5 | Increased blood, astrocyte and extracellular β-hydroxybutyrate; increased blood lactate; increased mito-NAD pool; increased initial concentration of coenzyme Q in neuron and astrocyte.  C_bHB_a = 3.3 mM (arterial bHB)  u0_ss[42] = (C_bHB_a/0.3)*0.2981203841045341 (capillary bHB initial value)  u0_ss[41] = (C_bHB_a/0.3)*0.002235 (astrocyte bHB, fixed)  u0_ss[40] = (C_bHB_a/0.3)*0.0028617229372728 (extracellular bHB)  C_Lac_a = 2.25 mM (arterial lactate)  u0_ss[149] = (C_Lac_a/0.75)*0.6887631395085969 (capillary lactate initial value)  NADtot = 1.1*0.000726 (mito neuron, astrocyte)  u0_ss[21] = 1.1*1.35  u0_ss[71] = 1.1*1.35 |
| th_172_blood_bhb_ini_dose1 | Slightly increased blood, astrocyte and extracellular β-hydroxybutyrate; slightly increased blood lactate; increased mito-NAD pool; increased initial concentration of coenzyme Q in neuron and astrocyte.  C_bHB_a = 0.5 mM (arterial bHB)  u0_ss[42] = (C_bHB_a/0.3)*0.2981203841045341 (capillary bHB initial value)  u0_ss[41] = (C_bHB_a/0.3)*0.002235 (astrocyte bHB, fixed)  u0_ss[40] = (C_bHB_a/0.3)*0.0028617229372728 (extracellular bHB)  C_Lac_a = 2.0 mM (arterial lactate)  u0_ss[149] = (C_Lac_a/0.75)*0.6887631395085969 (capillary lactate initial value)  NADtot = 1.1*0.000726 (mito neuron, astrocyte)  u0_ss[21] = 1.1*1.35  u0_ss[71] = 1.1*1.35 |
| th_173_blood_bhb_ini_dose2 | Increased blood, astrocyte and extracellular β-hydroxybutyrate; slightly increased blood lactate; increased mito-NAD pool; increased initial concentration of coenzyme Q in neuron and astrocyte.  C_bHB_a = 1.0 mM (arterial bHB)  u0_ss[42] = (C_bHB_a/0.3)*0.2981203841045341 (capillary bHB initial value)  u0_ss[41] = (C_bHB_a/0.3)*0.002235 (astrocyte bHB, fixed)  u0_ss[40] = (C_bHB_a/0.3)*0.0028617229372728 (extracellular bHB)  C_Lac_a = 2.0 mM (arterial lactate)  u0_ss[149] = (C_Lac_a/0.75)*0.6887631395085969 (capillary lactate initial value)  NADtot = 1.1*0.000726 (mito neuron, astrocyte)  u0_ss[21] = 1.1*1.35  u0_ss[71] = 1.1*1.35 |
| th_174_blood_bhb_ini_dose3 | Increased blood, astrocyte and extracellular β-hydroxybutyrate; slightly increased blood lactate; increased mito-NAD pool; increased initial concentration of coenzyme Q in neuron and astrocyte.  C_bHB_a = 2.0 mM (arterial bHB)  u0_ss[42] = (C_bHB_a/0.3)*0.2981203841045341 (capillary bHB initial value)  u0_ss[41] = (C_bHB_a/0.3)*0.002235 (astrocyte bHB, fixed)  u0_ss[40] = (C_bHB_a/0.3)*0.0028617229372728 (extracellular bHB)  C_Lac_a = 2.0 mM (arterial lactate)  u0_ss[149] = (C_Lac_a/0.75)*0.6887631395085969 (capillary lactate initial value)  NADtot = 1.1*0.000726 (mito neuron, astrocyte)  u0_ss[21] = 1.1*1.35  u0_ss[71] = 1.1*1.35 |
| th_175_blood_bhb_ini_dose4 | Increased blood, astrocyte and extracellular β-hydroxybutyrate; slightly increased blood lactate; increased mito-NAD pool; increased initial concentration of coenzyme Q in neuron and astrocyte.  C_bHB_a = 4.0 mM (arterial bHB)  u0_ss[42] = (C_bHB_a/0.3)*0.2981203841045341 (capillary bHB initial value)  u0_ss[41] = (C_bHB_a/0.3)*0.002235 (astrocyte bHB, fixed)  u0_ss[40] = (C_bHB_a/0.3)*0.0028617229372728 (extracellular bHB)  C_Lac_a = 2.0 mM (arterial lactate)  u0_ss[149] = (C_Lac_a/0.75)*0.6887631395085969 (capillary lactate initial value)  NADtot = 1.1*0.000726 (mito neuron, astrocyte)  u0_ss[21] = 1.1*1.35  u0_ss[71] = 1.1*1.35 |
| th_176_blood_bhb_ini_dose5 | Increased blood, astrocyte and extracellular β-hydroxybutyrate; slightly increased blood lactate; increased mito-NAD pool; increased initial concentration of coenzyme Q in neuron and astrocyte.  C_bHB_a = 8.0 mM (arterial bHB)  u0_ss[42] = (C_bHB_a/0.3)*0.2981203841045341 (capillary bHB initial value)  u0_ss[41] = (C_bHB_a/0.3)*0.002235 (astrocyte bHB, fixed)  u0_ss[40] = (C_bHB_a/0.3)*0.0028617229372728 (extracellular bHB)  C_Lac_a = 2.0 mM (arterial lactate)  u0_ss[149] = (C_Lac_a/0.75)*0.6887631395085969 (capillary lactate initial value)  NADtot = 1.1*0.000726 (mito neuron, astrocyte)  u0_ss[21] = 1.1*1.35  u0_ss[71] = 1.1*1.35 |
| th_177_AcCoAmito_na_ini_dose1 | Increased blood, astrocyte and extracellular β-hydroxybutyrate; slightly increased blood lactate; increased mito-NAD pool; increased initial concentration of coenzyme Q in neuron and astrocyte, increased AcCoA in neuron and astrocyte.  C_bHB_a = 3.3 mM (arterial bHB)  u0_ss[42] = (C_bHB_a/0.3)*0.2981203841045341 (capillary bHB initial value)  u0_ss[41] = (C_bHB_a/0.3)*0.002235 (astrocyte bHB, fixed)  u0_ss[40] = (C_bHB_a/0.3)*0.0028617229372728 (extracellular bHB)  C_Lac_a = 2.0 mM (arterial lactate)  u0_ss[149] = (C_Lac_a/0.75)*0.6887631395085969 (capillary lactate initial value)  NADtot = 1.1*0.000726 (mito neuron, astrocyte)  u0_ss[21] = 1.1*1.35  u0_ss[71] = 1.1*1.35  u0_ss[35] = 1.1*0.040486317761993  u0_ss[85] = 1.1*0.0042886443294129 |
| th_178_AcCoAmito_na_ini_dose2 | Increased blood, astrocyte and extracellular β-hydroxybutyrate; slightly increased blood lactate; increased mito-NAD pool; increased initial concentration of coenzyme Q in neuron and astrocyte, increased AcCoA in neuron and astrocyte.  C_bHB_a = 3.3 mM (arterial bHB)  u0_ss[42] = (C_bHB_a/0.3)*0.2981203841045341 (capillary bHB initial value)  u0_ss[41] = (C_bHB_a/0.3)*0.002235 (astrocyte bHB, fixed)  u0_ss[40] = (C_bHB_a/0.3)*0.0028617229372728 (extracellular bHB)  C_Lac_a = 2.0 mM (arterial lactate)  u0_ss[149] = (C_Lac_a/0.75)*0.6887631395085969 (capillary lactate initial value)  NADtot = 1.1*0.000726 (mito neuron, astrocyte)  u0_ss[21] = 1.1*1.35  u0_ss[71] = 1.1*1.35  u0_ss[35] = 1.2*0.040486317761993  u0_ss[85] = 1.2*0.0042886443294129 |
| th_179_AcCoAmito_na_ini_dose3 | Increased blood, astrocyte and extracellular β-hydroxybutyrate; slightly increased blood lactate; increased mito-NAD pool; increased initial concentration of coenzyme Q in neuron and astrocyte, increased AcCoA in neuron and astrocyte.  C_bHB_a = 3.3 mM (arterial bHB)  u0_ss[42] = (C_bHB_a/0.3)*0.2981203841045341 (capillary bHB initial value)  u0_ss[41] = (C_bHB_a/0.3)*0.002235 (astrocyte bHB, fixed)  u0_ss[40] = (C_bHB_a/0.3)*0.0028617229372728 (extracellular bHB)  C_Lac_a = 2.0 mM (arterial lactate)  u0_ss[149] = (C_Lac_a/0.75)*0.6887631395085969 (capillary lactate initial value)  NADtot = 1.1*0.000726 (mito neuron, astrocyte)  u0_ss[21] = 1.1*1.35  u0_ss[71] = 1.1*1.35  u0_ss[35] = 1.3*0.040486317761993  u0_ss[85] = 1.3*0.0042886443294129 |
| th_180_AcCoAmito_na_ini_dose4 | Increased blood, astrocyte and extracellular β-hydroxybutyrate; slightly increased blood lactate; increased mito-NAD pool; increased initial concentration of coenzyme Q in neuron and astrocyte, increased AcCoA in neuron and astrocyte.  C_bHB_a = 3.3 mM (arterial bHB)  u0_ss[42] = (C_bHB_a/0.3)*0.2981203841045341 (capillary bHB initial value)  u0_ss[41] = (C_bHB_a/0.3)*0.002235 (astrocyte bHB, fixed)  u0_ss[40] = (C_bHB_a/0.3)*0.0028617229372728 (extracellular bHB)  C_Lac_a = 2.0 mM (arterial lactate)  u0_ss[149] = (C_Lac_a/0.75)*0.6887631395085969 (capillary lactate initial value)  NADtot = 1.1*0.000726 (mito neuron, astrocyte)  u0_ss[21] = 1.1*1.35  u0_ss[71] = 1.1*1.35  u0_ss[35] = 1.4*0.040486317761993  u0_ss[85] = 1.4*0.0042886443294129 |
| th_181_AcCoAmito_na_ini_dose5 | Increased blood, astrocyte and extracellular β-hydroxybutyrate; slightly increased blood lactate; increased mito-NAD pool; increased initial concentration of coenzyme Q in neuron and astrocyte, increased AcCoA in neuron and astrocyte.  C_bHB_a = 3.3 mM (arterial bHB)  u0_ss[42] = (C_bHB_a/0.3)*0.2981203841045341 (capillary bHB initial value)  u0_ss[41] = (C_bHB_a/0.3)*0.002235 (astrocyte bHB, fixed)  u0_ss[40] = (C_bHB_a/0.3)*0.0028617229372728 (extracellular bHB)  C_Lac_a = 2.0 mM (arterial lactate)  u0_ss[149] = (C_Lac_a/0.75)*0.6887631395085969 (capillary lactate initial value)  NADtot = 1.1*0.000726 (mito neuron, astrocyte)  u0_ss[21] = 1.1*1.35  u0_ss[71] = 1.1*1.35  u0_ss[35] = 1.5*0.040486317761993  u0_ss[85] = 1.5*0.0042886443294129 |
| th_182_SUCCOAmito_na_ini_dose1 | Increased blood, astrocyte and extracellular β-hydroxybutyrate; slightly increased blood lactate; increased mito-NAD pool; increased initial concentration of coenzyme Q in neuron and astrocyte, increased SucCoA in neuron and astrocyte.  C_bHB_a = 3.3 mM (arterial bHB)  u0_ss[42] = (C_bHB_a/0.3)*0.2981203841045341 (capillary bHB initial value)  u0_ss[41] = (C_bHB_a/0.3)*0.002235 (astrocyte bHB, fixed)  u0_ss[40] = (C_bHB_a/0.3)*0.0028617229372728 (extracellular bHB)  C_Lac_a = 2.0 mM (arterial lactate)  u0_ss[149] = (C_Lac_a/0.75)*0.6887631395085969 (capillary lactate initial value)  NADtot = 1.1*0.000726 (mito neuron, astrocyte)  u0_ss[21] = 1.1*1.35  u0_ss[71] = 1.1*1.35  u0_ss[29] = 1.1*0.0027652024434962  u0_ss[79] = 1.1*0.0016699868314113 |
| th_183_SUCCOAmito_na_ini_dose2 | Increased blood, astrocyte and extracellular β-hydroxybutyrate; slightly increased blood lactate; increased mito-NAD pool; increased initial concentration of coenzyme Q in neuron and astrocyte, increased SucCoA in neuron and astrocyte.  C_bHB_a = 3.3 mM (arterial bHB)  u0_ss[42] = (C_bHB_a/0.3)*0.2981203841045341 (capillary bHB initial value)  u0_ss[41] = (C_bHB_a/0.3)*0.002235 (astrocyte bHB, fixed)  u0_ss[40] = (C_bHB_a/0.3)*0.0028617229372728 (extracellular bHB)  C_Lac_a = 2.0 mM (arterial lactate)  u0_ss[149] = (C_Lac_a/0.75)*0.6887631395085969 (capillary lactate initial value)  NADtot = 1.1*0.000726 (mito neuron, astrocyte)  u0_ss[21] = 1.1*1.35  u0_ss[71] = 1.1*1.35  u0_ss[29] = 1.2*0.0027652024434962  u0_ss[79] = 1.2*0.0016699868314113 |
| th_184_SUCCOAmito_na_ini_dose3 | Increased blood, astrocyte and extracellular β-hydroxybutyrate; slightly increased blood lactate; increased mito-NAD pool; increased initial concentration of coenzyme Q in neuron and astrocyte, increased SucCoA in neuron and astrocyte.  C_bHB_a = 3.3 mM (arterial bHB)  u0_ss[42] = (C_bHB_a/0.3)*0.2981203841045341 (capillary bHB initial value)  u0_ss[41] = (C_bHB_a/0.3)*0.002235 (astrocyte bHB, fixed)  u0_ss[40] = (C_bHB_a/0.3)*0.0028617229372728 (extracellular bHB)  C_Lac_a = 2.0 mM (arterial lactate)  u0_ss[149] = (C_Lac_a/0.75)*0.6887631395085969 (capillary lactate initial value)  NADtot = 1.1*0.000726 (mito neuron, astrocyte)  u0_ss[21] = 1.1*1.35  u0_ss[71] = 1.1*1.35  u0_ss[29] = 1.3*0.0027652024434962  u0_ss[79] = 1.3*0.0016699868314113 |
| th_185_SUCCOAmito_na_ini_dose4 | Increased blood, astrocyte and extracellular β-hydroxybutyrate; slightly increased blood lactate; increased mito-NAD pool; increased initial concentration of coenzyme Q in neuron and astrocyte, increased SucCoA in neuron and astrocyte.  C_bHB_a = 3.3 mM (arterial bHB)  u0_ss[42] = (C_bHB_a/0.3)*0.2981203841045341 (capillary bHB initial value)  u0_ss[41] = (C_bHB_a/0.3)*0.002235 (astrocyte bHB, fixed)  u0_ss[40] = (C_bHB_a/0.3)*0.0028617229372728 (extracellular bHB)  C_Lac_a = 2.0 mM (arterial lactate)  u0_ss[149] = (C_Lac_a/0.75)*0.6887631395085969 (capillary lactate initial value)  NADtot = 1.1*0.000726 (mito neuron, astrocyte)  u0_ss[21] = 1.1*1.35  u0_ss[71] = 1.1*1.35  u0_ss[29] = 1.4*0.0027652024434962  u0_ss[79] = 1.4*0.0016699868314113 |
| th_186_SUCCOAmito_na_ini_dose5 | Increased blood, astrocyte and extracellular β-hydroxybutyrate; slightly increased blood lactate; increased mito-NAD pool; increased initial concentration of coenzyme Q in neuron and astrocyte, increased SucCoA in neuron and astrocyte.  C_bHB_a = 3.3 mM (arterial bHB)  u0_ss[42] = (C_bHB_a/0.3)*0.2981203841045341 (capillary bHB initial value)  u0_ss[41] = (C_bHB_a/0.3)*0.002235 (astrocyte bHB, fixed)  u0_ss[40] = (C_bHB_a/0.3)*0.0028617229372728 (extracellular bHB)  C_Lac_a = 2.0 mM (arterial lactate)  u0_ss[149] = (C_Lac_a/0.75)*0.6887631395085969 (capillary lactate initial value)  NADtot = 1.1*0.000726 (mito neuron, astrocyte)  u0_ss[21] = 1.1*1.35  u0_ss[71] = 1.1*1.35  u0_ss[29] = 1.5*0.0027652024434962  u0_ss[79] = 1.5*0.0016699868314113 |
| th_187_ATP_dose1 | Increased cytosolic ATP pool, initial concentration of ATP in the neuron and neuronal IMS.  ATDPtot_n = 1.5  u0_ss[23] = 1.4  u0_ss[13] = 1.4  (Rangaraju et al., 2014; Baeza-Lehnert et al., 2019) |
| th_188_ATP_dose2 | Increased cytosolic ATP pool, initial concentration of ATP in the neuron and neuronal IMS.  ATDPtot_n = 1.525  u0_ss[23] = 1.425  u0_ss[13] = 1.425 |
| th_189_ATP_dose3 | Increased cytosolic ATP pool, initial concentration of ATP in the neuron and neuronal IMS.  ATDPtot_n = 1.55  u0_ss[23] = 1.45  u0_ss[13] = 1.45 |
| th_190_ATP_dose4 | Increased cytosolic ATP pool, initial concentration of ATP in the neuron and neuronal IMS.  ATDPtot_n = 1.575  u0_ss[23] = 1.475  u0_ss[13] = 1.475 |
| th_191_ATP_dose5 | Increased cytosolic ATP pool, initial concentration of ATP in the neuron and neuronal IMS.  ATDPtot_n = 1.6  u0_ss[23] = 1.5  u0_ss[13] = 1.5 |

**Supplemental References**

Baeza-Lehnert F, Saab AS, Gutierrez R,…Barros LF (2019) Non-Canonical Control of Neuronal Energy Status by the Na^+^ Pump. Cell Metabolism, Volume 29, Issue 3, 668 - 680.e4 doi: 10.1016/j.cmet.2018.11.005

### Simmons, RA (2017) Cell Glucose Transport and Glucose Handling During Fetal and Neonatal Development, in Fetal and Neonatal Physiology, 5th Ed., Polin, Abman, Rowitch, Benitz, Fox editors. Elsevier Inc. ISBN 978-0-323-35214-7. <https://doi.org/10.1016/B978-0-323-35214-7.00043-3>.

Klepper J, Akman C, Armeno M, Auvin S, Cervenka M, Cross HJ, De Giorgis V, Della Marina A, Engelstad K, Heussinger N, Kossoff EH, Leen WG, Leiendecker B, Monani UR, Oguni H, Neal E, Pascual JM, Pearson TS, Pons R, Scheffer IE, Veggiotti P, Willemsen M, Zuberi SM, De Vivo DC. (2020) Glut1 Deficiency Syndrome (Glut1DS): State of the art in 2020 and recommendations of the international Glut1DS study group. Epilepsia Open. 2020 Aug 13;5(3):354-365. doi: 10.1002/epi4.12414. PMID: 32913944; PMCID: PMC7469861.

Rangaraju V, Calloway N, Ryan TA (2014) Activity-Driven Local ATP Synthesis Is Required for Synaptic Function Cell, Volume 156, Issue 4, 825 – 835. doi: 10.1016/j.cell.2013.12.042.

Rajasekaran K, Ma Q, Good LB, Kathote G, Jakkamsetti V, Liu P, Avila A, Primeaux S, Enciso Alva J, Markussen KH, Marin-Valencia I, Sirsi D, Hacker PMS, Gentry MS, Su J, Lu H, Pascual JM. (2022) Metabolic modulation of synaptic failure and thalamocortical hypersynchronization with preserved consciousness in Glut1 deficiency. Sci Transl Med. 2022 Oct 5;14(665):eabn2956. doi: 10.1126/scitranslmed.abn2956. Epub 2022 Oct 5. PMID: 36197967; PMCID: PMC10276203.

Wang, D., Yang, H., Shi, L. *et al.* (2008) Functional Studies of the T295M Mutation Causing Glut1 Deficiency: Glucose Efflux Preferentially Affected by T295M. *Pediatr Res* 64, 538–543 (2008). https://doi.org/10.1203/PDR.0b013e318184d2b5

**Table B in S1 Text**

**Metadata for Figures:**

Values from graphs including means, standard deviations

**Fig 1**

C

Means:


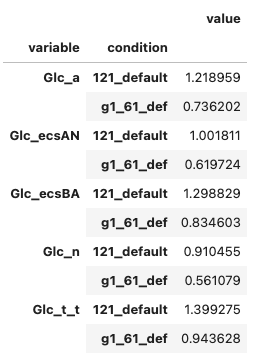


Stds:


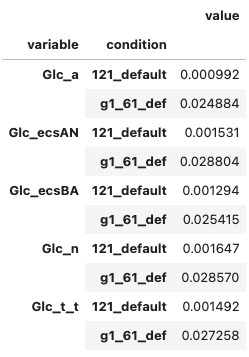


**Fig2**

A - control

Means:


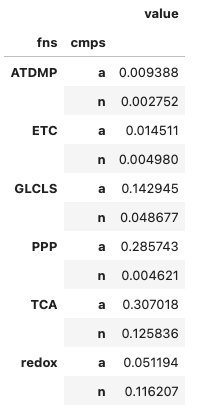


Stds:


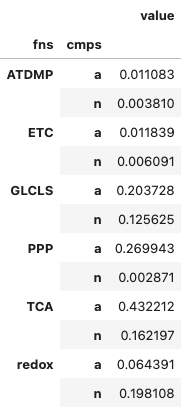


A - GLUT1-def

Means:


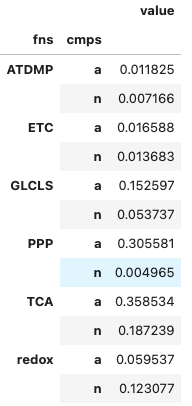


Stds:


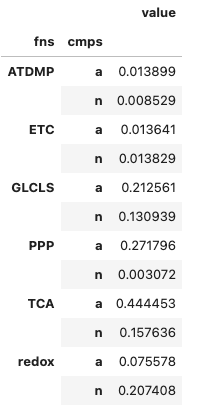


B - control

Vertical lines shown on the figure:

dotted, color=#208c48 -2.706261105396236

dotted, color=#03402e -1.825968282365485

solid line, color=#208c48 -5.156003957815724

solid line, color=#03402e -3.598420598810139

B - GLUT1-def

Vertical lines shown on the figure:

dotted, color=red -2.4219826258117956

dotted, color=darkred -1.703862127178729

solid line, color=red -4.38752324980128

solid line, color=darkred -3.3356663771048494

C

Means:


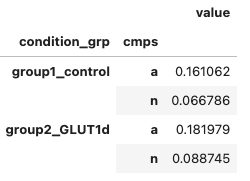


Stds:


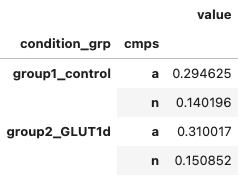


**Fig 3**

C


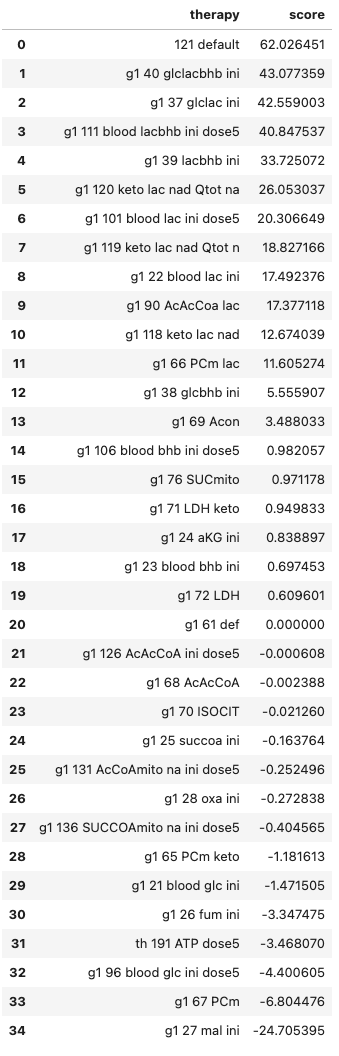


D


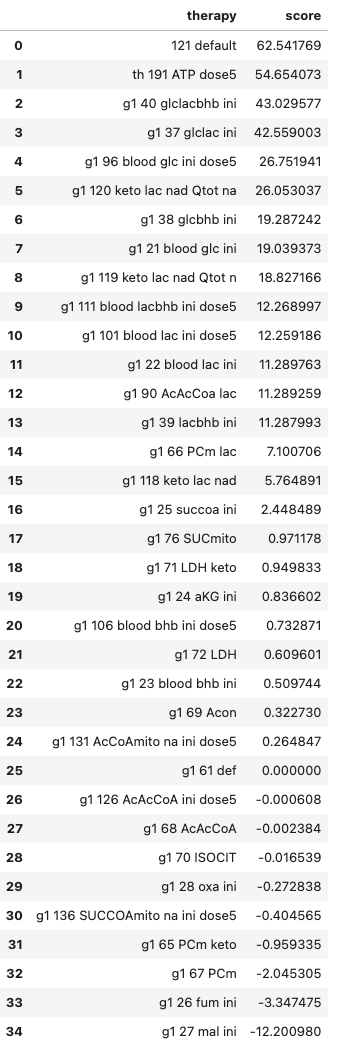


**Supplementary Figures**

**Fig S2**

Glc

Means:


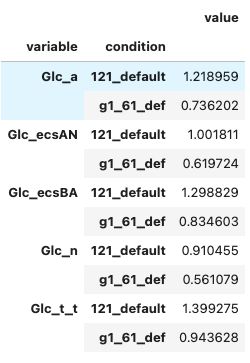


Stds:


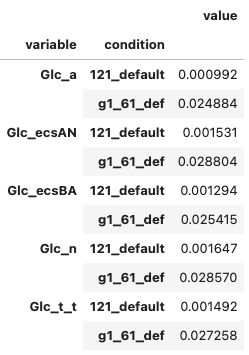


FBP

Means:


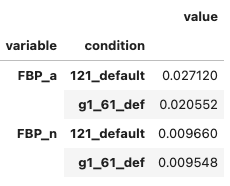


Stds:


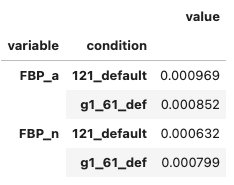


Lac

Means:


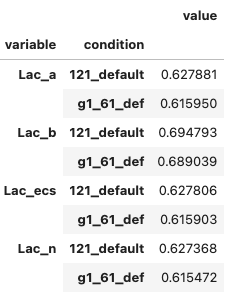


Stds:


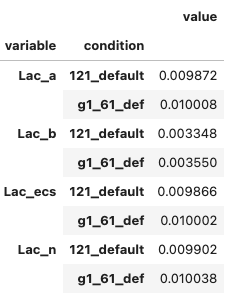


DHAP,GAP

Means:


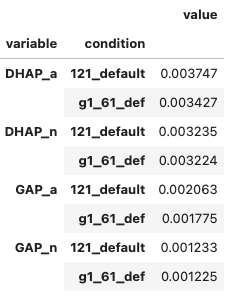


Stds:


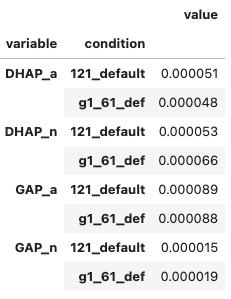


OXAmito, SUCCOAmito

Means:


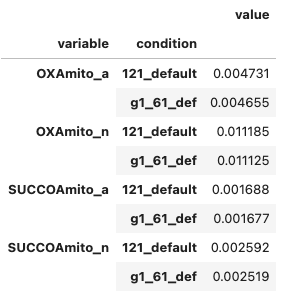


Stds:


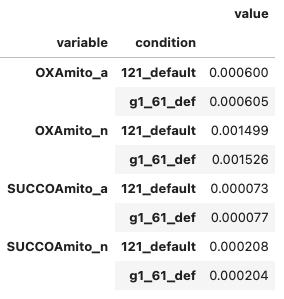


AKGmito, FUMmito

Means:


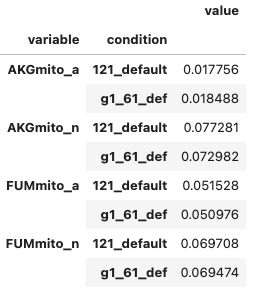


Stds:


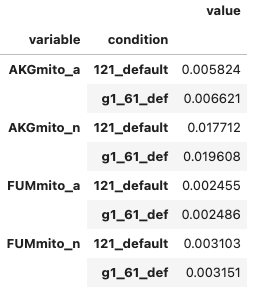


AcCoAmito, CoAmito, ISOCITmito, PYRmito

Means:


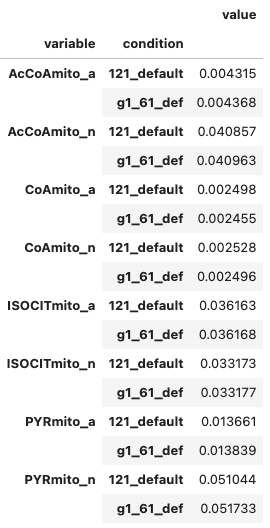


Stds:


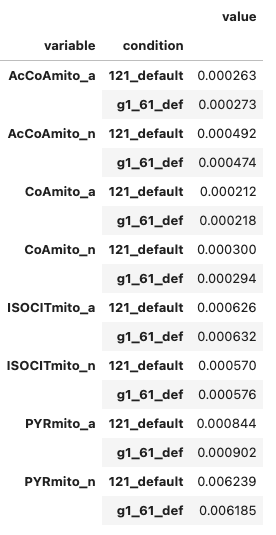


CITmito, MALmito, SUCmito

Means:


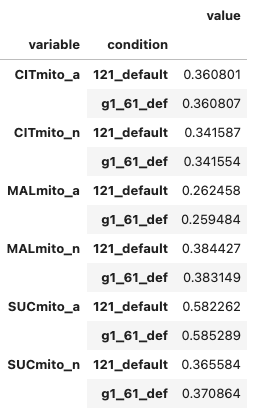


Stds:


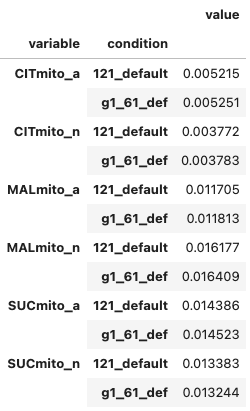


**Fig S3**

1. Means:


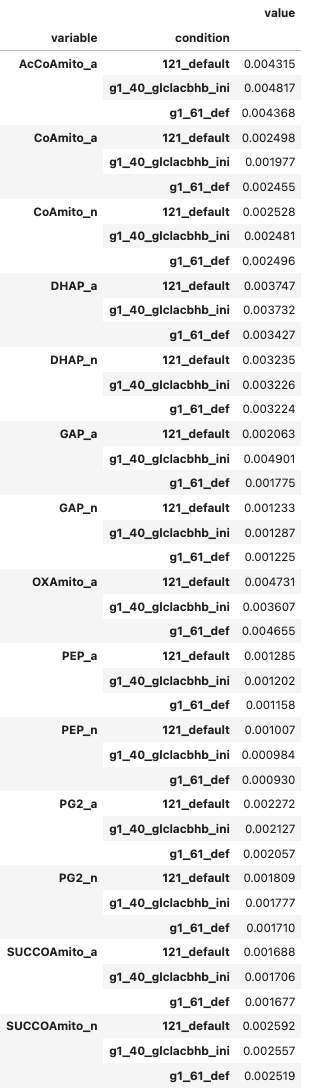


Stds:


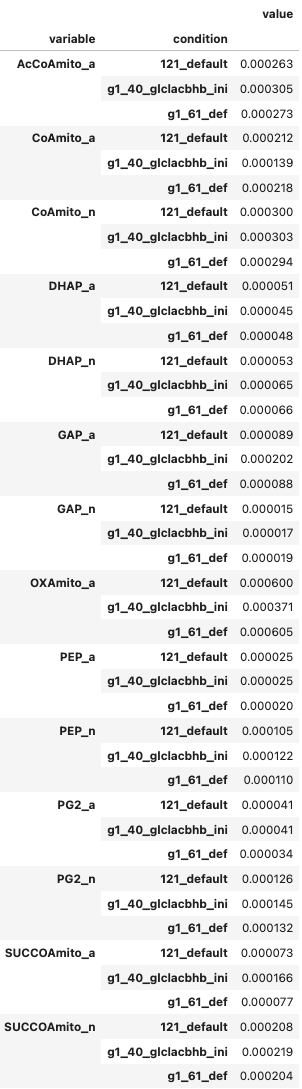


1. Means:


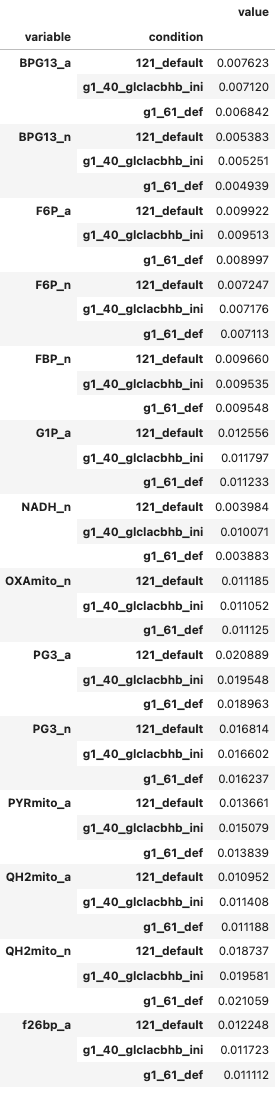


Stds:


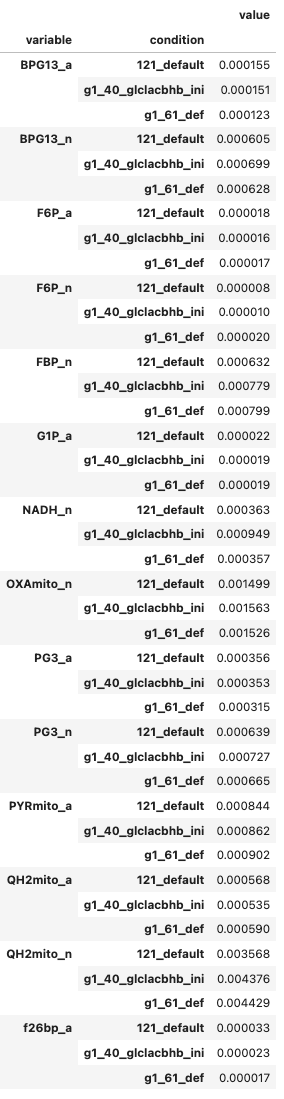


1. Means:


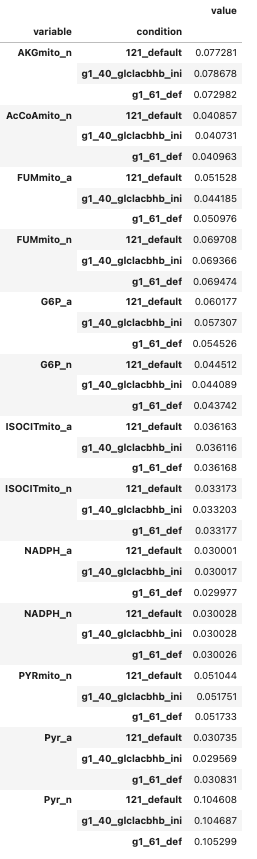


Stds:


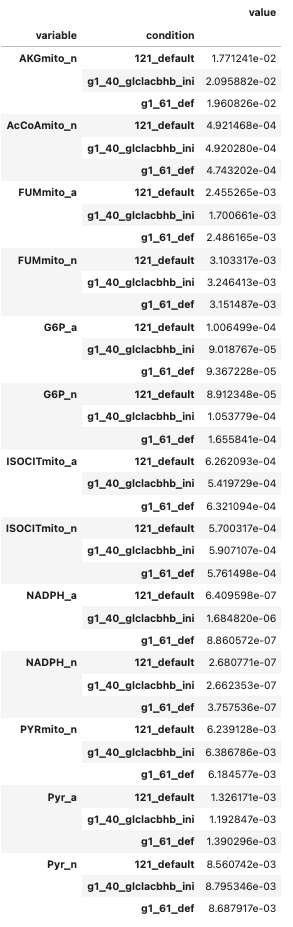


1. Means:


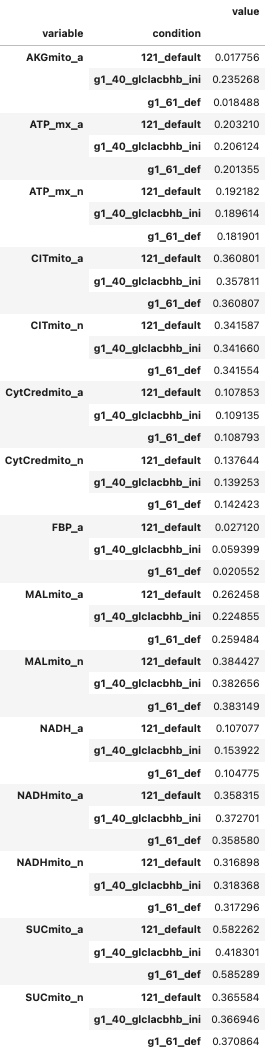


Stds:


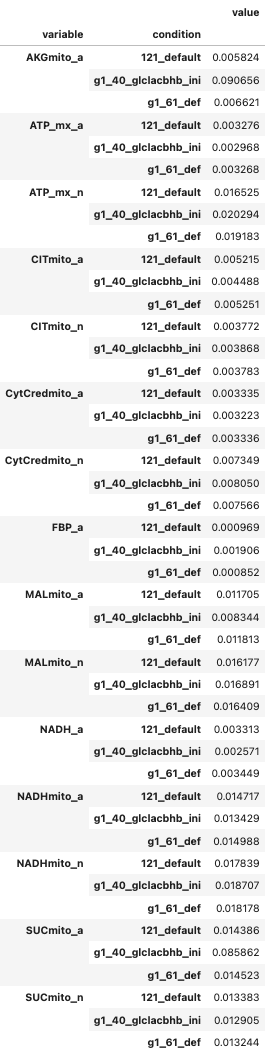


1. Means:


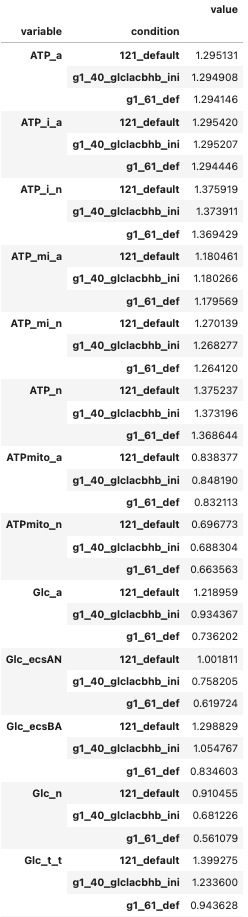

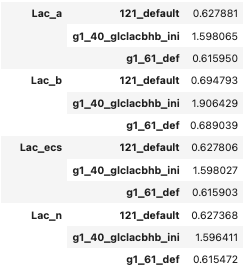


Stds:


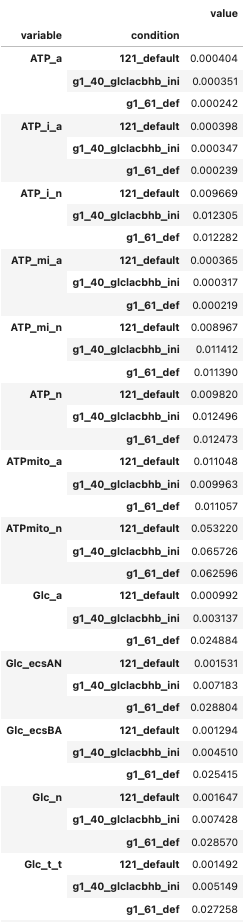

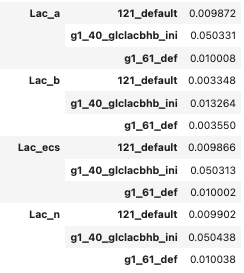


6) Means:


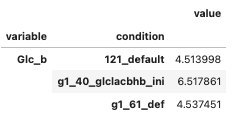


Stds:


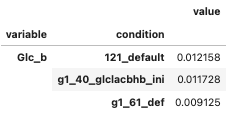

Supplement: S1 Text — (ZIP) [file pcbi.1012959.s001.docx]
